# Cis-regulatory analysis of Onecut1 expression in fate-restricted retinal progenitor cells

**DOI:** 10.1186/s13064-020-00142-w

**Published:** 2020-03-19

**Authors:** Sruti Patoori, Nathalie Jean-Charles, Ariana Gopal, Sacha Sulaiman, Sneha Gopal, Brian Wang, Benjamin Souferi, Mark M. Emerson

**Affiliations:** 1 Biology PhD Program, The Graduate Center, The City University of New York, New York, NY 10016 USA; 2 Department of Biology, The City College of New York, The City University of New York, New York, NY 10031 USA; 3 Present Address: Doctoral program in Department of Chemical and Biological Engineering, Center for Biotechnology and Interdisciplinary Studies, Rensselaer Polytechnic Institute, Troy, NY 12180 USA; 4 Present Address: Touro College of Osteopathic Medicine, New York, NY 10027 USA; 5 Biochemistry PhD Program, Graduate Center, City University of New York, New York, NY 10016 USA

**Keywords:** Retinal progenitor cells, Multipotent, Fate-restricted, Cone photoreceptors, Basic helix-loop-helix, Chicken, Electroporation

## Abstract

**Background:**

The vertebrate retina consists of six major classes of neuronal cells. During development, these cells are generated from a pool of multipotent retinal progenitor cells (RPCs) that express the gene Vsx2. Fate-restricted RPCs have recently been identified, with limited mitotic potential and cell fate possibilities compared to multipotent RPCs. One population of fate-restricted RPCs, marked by activity of the regulatory element ThrbCRM1, gives rise to both cone photoreceptors and horizontal cells. These cells do not express Vsx2, but co-express the transcription factors (TFs) Onecut1 and Otx2, which bind to ThrbCRM1. The components of the gene regulatory networks that control the transition from multipotent to fate-restricted gene expression are not known. This work aims to identify and evaluate cis-regulatory elements proximal to Onecut1 to identify the gene regulatory networks involved in RPC fate-restriction.

**Method:**

We identified regulatory elements through ATAC-seq and conservation, followed by reporter assays to screen for activity based on temporal and spatial criteria. The regulatory elements of interest were subject to deletion and mutation analysis to identify functional sequences and evaluated by quantitative flow cytometry assays. Finally, we combined the enhancer::reporter assays with candidate TF overexpression to evaluate the relationship between the TFs, the enhancers, and early vertebrate retinal development. Statistical tests included ANOVA, Kruskal-Wallis, or unpaired t-tests.

**Results:**

Two regulatory elements, ECR9 and ECR65, were identified to be active in ThrbCRM1(+) restricted RPCs. Candidate bHLH binding sites were identified as critical sequences in both elements. Overexpression of candidate bHLH TFs revealed specific enhancer-bHLH interactions. Nhlh1 overexpression expanded ECR65 activity into the Vsx2(+) RPC population, and overexpression of NeuroD1/NeuroG2/NeuroD4 had a similar effect on ECR9. Furthermore, bHLHs that were able to activate ectopic ECR9 reporter were able to induce endogenous Otx2 expression.

**Conclusions:**

This work reports a large-scale screen to identify spatiotemporally specific regulatory elements near the Onecut1 locus. These elements were used to identify distinct populations in the developing retina. In addition, fate-restricted regulatory elements responded differentially to bHLH factors, and suggest a role for retinal bHLHs upstream of the Otx2 and Onecut1 genes during the formation of restricted RPCs from multipotent RPCs.

## Introduction

The vertebrate retina is comprised of six main classes of neuronal cells and one class of glial cells, organized into three discrete nuclear layers and two plexiform layers. These morphologically and functionally diverse cells have been characterized in multiple vertebrate species to originate from multipotent retinal progenitor cells (RPCs) [12, 41]. The vertebrate retina is therefore a valuable model to study neuronal cell fate choice. The process of retinal development is highly conserved throughout the vertebrate subphylum, with regards to the birth order of the various cell types [44, 47] and the developmental regulatory networks involved. However, the gene regulatory networks (GRNs) that mediate the generation of specific restricted RPCs from multipotent RPCs are largely unknown, as are the networks that function in restricted RPCs to define their fate potential.

One restricted RPC type that has been identified across zebrafish, chick, and mouse models preferentially generates cones and horizontal cells (HCs) and has been identified in zebrafish and chick through regulatory elements associated with the Thrb and Olig2 genes [11, 16, 39]. For example, analysis in zebrafish and mouse RPCs showed that the same RPC can give rise to both cone and horizontal cell precursor cells [16, 39]. Endogenous Thrb expression has been observed in Otx2-expressing early RPCs in the chick, suggesting that reporters driven by regulatory elements correspond to in vivo regulatory events in the retina [40]. While Otx2 expression is involved in multiple cell fates during retinal development, it has been shown that the combination of Otx2 and Onecut1 activates ThrbCRM1, which is a specific Thrb cis-regulatory element (CRE) active in cone/HC restricted RPCs (RPC [CH]) [11]. Loss-of-function mutations in Otx2 and Onecut1 affect early cone gene expression, cone number, cone type, and horizontal cell genesis [31, 35, 45], suggesting that these transcription factors (TFs) are critical in the gene regulatory networks of ThrbCRM1 restricted RPCs.

The population of restricted RPCs marked by ThrbCRM1 have been shown to be molecularly distinct from multipotent RPCs. ThrbCRM1(+) RPCs downregulate multipotent RPC genes such as Vsx2 while Onecut1 is upregulated [6]. Onecut1 expression is further upregulated in the HC progeny of these cells but is downregulated in the cone photoreceptor progeny. However, it is not known how Onecut1 expression is activated in the ThrbCRM1 RPC population or what distinguishes it from other Onecut1(+) cell populations.

The regulatory module that connects Otx2 and Onecut1 to Thrb expression demonstrates the importance of both cis- and trans- regulatory elements in directing retinal cell fate. Cell fate specification and fate restriction require the combinatorial expression of multiple developmental transcription factors. As such, cell-type specific cis-regulatory elements can define the intermediate/restricted RPCs that may be difficult to identify through only the transient expression of developmental transcription factors that are often involved in the specification of multiple retinal cell types. These regulatory elements can be used to facilitate imaging, lineage tracing, and molecular analysis, while also providing insights into the relationships between RPC populations.

We sought to identify the cis-regulatory elements upstream of Onecut1 expression in cone/HC restricted RPCs and to determine the transcription factors that occupy these elements. To this end, we conducted a multi-step screen to identify Onecut1-associated non-coding DNA elements capable of driving reporter transcription in early retinal RPCs that give rise to cones and horizontal cells in the early embryonic chick retina. The candidate regulatory elements that emerged from the screen were then bioinformatically analyzed for transcription factor binding sites. Mutational analyses facilitated the functional evaluation of these predicted TF binding sites. Overexpression experiments were used to determine the relationship between the predicted transcription factors and Onecut1 expression. We identified two regulatory elements, ECR9 and ECR65, located upstream of the Onecut1 coding region. These both contain predicted binding sites for bHLH transcription factors, which are known to be functionally important for retinal development. We show that both of these elements require the predicted bHLH binding sites for their activity and that each element responds to distinct bHLH factors, the transcripts of which are enriched or present in the ThrbCRM1 population. Finally, we show that NeuroD1, NeuroG2, and NeuroD4 are sufficient to induce expression of Otx2 and that all four TFs including Nhlh1 are able to induce the activity of their corresponding regulatory elements in Vsx2(+) multipotent RPCs. Ultimately, this work further clarifies components of the gene regulatory network leading to the early retinal cell fates of cone photoreceptors, horizontal cells, and retinal ganglion cells.

## Methods

### Animals

Fertilized chick eggs were acquired from Charles River and stored at 16 °C room for a maximum of ten days. Embryonic days were counted from E0 when eggs were moved to a 38 °C humidified incubator for five days.

### ATAC-seq

ATAC-seq libraries were collected and amplified as outlined by [7]. Mouse retinas were collected from embryonic day 12.5 (plug morning equal to time 0.5) embryos, and dissociated using manual douncing. Libraries were analyzed for quality control on Bioanalyzer and Qubit and then sequenced at a depth of 37.5 million reads per sample. Sequenced libraries were prepared for analysis using FASTQGroomer [5] on default settings and then analyzed using Bowtie for Illumina [23] with default settings except -X 2000 and – m 1, through the usegalaxy.org [1] web platform. Resulting SAM files were converted to the BAM format [25] and the BigWig [22] format.

### Electroporation

Retinae were electroporated ex vivo as previously described [36]. CAG::reporter plasmids were used at a concentration of 0.1μg/uL with the exception of CAG::mCherry, which was used at a concentration of 0.04 μg/uL. Reporter plasmids under the control of a tissue-specific enhancer (ex: ThrbCRM1, ECR9, ACR2, etc) were used at a concentration of 0.16 μg/uL. For lineage tracing experiments, all plasmids (recombinase, responder plasmid, co-electroporation control) were used at a concentration of 0.1 μg/uL. Recombinase and responder plasmids are described in [36]. Plasmids used in initial identification screen for cis-regulatory elements used miniprep scale DNA purification (Zymo Research, D4020 or 5 prime/Eppendorf, FastPlasmid kit) and all subsequent experiments used midiprep scale DNA purification (Qiagen, 12145 or 12243).

### Alkaline phosphatase staining

Retinas were harvested from culture and fixed in 4% paraformaldehyde (PFA), washed 3X in PBS, and incubated in 1 mL NTM (pH 9.5) buffer for 15 min while shaking at a low speed before addition of 1 mL NTM with NBT (0.25 mg/mL) and BCIP (0.125 mg/mL). Retinae were incubated with the AP substrates in the dark for 2–3 h until the positive control was well-stained.

### Immunohistochemistry

Retinas were harvested from culture, prepared for cryosectioning, and 20 μm vertical sections were collected as outlined in [36]. Sections were incubated for 10 min in 0.1% Tween (VWR, 97062–332) in PBS (PBT) and then blocked for 1 h in 5% serum (Jackson ImmunoResearch, Donkey - 017-000121, Goat - 005-000-121) in PBT at room temperature prior to incubation with primary antibodies overnight at 4 C. Sections were washed 3X with PBT, prior to blocking at room temperature for 30 min and then incubated with secondary antibodies overnight at 4 C. DAPI was added at 1 μg/uL in PBT while washing off the secondary antibodies. Sections were then mounted using Flouromount-G (Southern Biotech, 0100–01) and coverslips (VWR, 48393–106). Primary antibodies are listed in the table below. All secondary antibodies were obtained from Jackson ImmunoResearch and suitable for multiple labelling. All Alexa-conjugated secondary antibodies were used at dilution of 1:400 and Cy3-conjugated secondary antibodies were used at a dilution of 1:250 from secondary antibody stocks in 50% glycerol.

**Table:** 

Antibody; dilution	Vendor	Catalog number
Chick anti-GFP 1:2000	Abcam	ab13970
Rabbit anti-GFP 1:1000	Invitrogen	A6455
Mouse anti-AU1 1:2000	Enzo	ENZ-ABS135–0200
Chick anti-Bgal 1:1000	Abcam	ab9361
Rabbit anti-RFP 1:250	Rockland	600–401-379
Mouse anti-Onecut1 1:200	Santa Cruz	Sc-376308
Goat anti-Otx2 1:500	R&D	AF1979
Sheep anti-Vsx2 1:200	Ex Alpha	x1180p
Mouse anti-Visinin 1:250	DHSB	7G4-s
Mouse anti-Lim1 1:15	DHSB	4F2-C
Mouse anti-Isl1 1:50	DHSB	39.3F7
Mouse anti-panBrn3 1:100	Santa Cruz	Sc-390781

### Microscopy and cell counting

Images of whole AP-stained retinas were acquired using a Zeiss Axiozoom V16 microscope with a 1X objective and Zen 2 Blue 2011 software. All confocal microscopy images were acquired using a Zeiss LSM 710 inverted confocal microscope with a 40x oil immersion objective, 488 nm laser, 561 nm laser, 633 nm laser, 405 nm laser, and Zen Black 2015 21 SP2 software at a resolution of 1024 × 1024, acquisition speed of 6 and averaging number of 2. For all confocal images shown, Z-stacks consisting of 8–12 Z planes were collected and are shown as maximum intensity projections. Cells were counted in the Fiji [37] distribution of ImageJ, using the Cell Counter plug-in developed by Kurt De Vos. In the lineage-tracing experiments and for the EdU assay, all GFP(+) cells which co-localized with DAPI were counted first and cells positive for EdU or for cell-specific markers such as Visinin were counted among this population. In both instances, multiple images per retina were analyzed if necessary to reach at least 45–50 GFP(+) cells. Brightness and contrast were adjusted uniformly across each image in Affinity Designer vector editor (Serif [Europe] Ltd).

### EdU pulse and detection

5 uL of 10 mM EdU solution was added to 1 mL of culture media during the last hour of incubation before retinas are harvested as described above. EdU was detected using the Click-iT Plus EdU Kit for Imaging (Invitrogen, C10640). The tissue was first incubated for 15 min in 0.1% Tween in PBS at room temperature before incubating with the EdU Reaction Cocktail for 30 min in the dark at room temperature. The EdU Reaction Cocktail was then removed with 3 washes of PBT prior to antibody staining.

### Deletions and mutagenesis

Deletions or truncated versions of regulatory element sequences were generated through the use of PCR primers that began internally within the sequence and excluded the portions of the sequence to be deleted. Mutant regulatory element sequences were generated using overlap extension PCR, in which primers included short sequence mismatches at potential TF binding sites. A second set of primers encompassed the ends of the regulatory element sequence and the restriction sites within the plasmid template for easy cloning of the mutant sequence into the Stagia3 reporter vector. Site 3 and Site 4 within ECR9 were mutated to the sequences “TTAGAC” and “AGGCCA”. The bHLH site within ECR65 Region 4 was also mutated twice, to the sequence “CTGATGAATGGCG” to include the E-box site, 4 bp upstream and 3 bp downstream and to the sequence “TTTCCCAAAG” to include the E-box site and 4 bp upstream. (Fig. 4, Additional File 8).

### MEME-suite

To identify conserved motifs within ECR65, we used MEME with settings to find a maximum of 12 motifs, with a width of 6–50 bp each and 2–9 sites per motif. For ECR9, we used settings to find a maximum of 7 motifs, with a width of 6–50 bp each and 2–8 sites per motif. The output from MEME was then used as input for TOMTOM under default settings.

### Multiple sequence alignments

The alignments between the chick, mouse, and human sequences for ECR9 and ECR65 (Additional File 7) were produced in Clustal Omega [27] version 1.2.4 with default settings.

### Plasmids

Plasmids containing coding sequences of candidate TF genes were obtained from Transomics. Mouse Nhlh1 (Clone ID: BC051018) and mouse NeuroD4 (Clone ID: BC054391) were cloned using EcoR1 to insert into a modified pCAG vector that allows for EcoR1 flanked insert cloning [11] while mouse NeuroD1 (Clone ID: BC018241), human NeuroG2 (Clone ID: BC036847) and human Atoh7 (Clone ID: BC032621) were cloned using a combination of EcoR1 and Not1 (NeuroD1, NeuroG2, Atoh7) into pCAG::EGFP [29] such that each coding sequence is under the control of the CAG promoter. The following plasmids were previously reported: CAG::OC1, ThrbCRM1::GFP, ThrbCRM1::AU1 plasmids [11]; CAG::iRFP [6]; Bp::PhiC31 lineage tracing and CAaNa::GFP responder plasmids [36]; UbiC::TdTomato [34]; and TdTomato reporter plasmid [19]. The CAG::mCherry and CAG::nucBgal plasmids were constructed by Takahiko Matsuda and reported in [43] and obtained from the Cepko lab, respectively. The ThrbCRM1::TdTomato plasmid was made by ligating a Not1/EcoR1 fragment from ThrbCRM1::GFP into the TdTomato reporter plasmid [19]. Candidate Onecut1 cis-regulatory elements were amplified from chick or mouse genomic DNA with Herculase II polymerase (Agilent, 600677–51), treated for 10–30 min with Taq polymerase (Qiagen, 201203) to generate Adenine overhangs, and ligated into PGemTeasy (Promega, A1360). Inserts were sequence verified by Sanger sequencing (Genewiz) and moved into Stagia3 after EcoR1 digestion. In cases where elements contained EcoR1 sites, the original sequences used to amplify candidate elements were used to generate modified oligos with Xho1, Sal1, or Mfe1 restriction sites to allow for PCR-amplification of elements from the verified PGemTeasy clones and subsequent insertion into an appropriately digested Stagia3 plasmid. As candidate elements could be inserted into Stagia3 in two orientations, for some elements both possible orientations were tested.

### Dissociation and flow Cytometry

Upon harvest, retinae were dissociated into single cells as described in [36] using papain (Worthington, LS003126) and an activation solution of L-cysteine (VWR, 97063–478) and 10 mM EDTA at 37 C. 10% FBS (ThermoFisher, A3160602) solution in DMEM (Life Technologies, 11995–073) was used to stop the dissociation. Cells were further digested with DNaseI (Sigma, 4536282001) and subsequently washed in DMEM prior to fixing in 4% PFA. Dissociated cells were then analyzed on a BD LSRII machine using the 488 nm, 561 nm, and 633 nm lasers. The collected data was analyzed using FlowJo version 10.4.2.

### Statistical tests

Statistical tests were conducted using GraphPad Prism. Data sets were tested for normality (Shapiro-Wilk) prior to ANOVA, Kruskal-Wallis, or t-tests. Significant Kruskal-Wallis or ANOVA *p*-values were followed up with Dunn’s or Dunnett’s post hoc test, respectively.

**Table:** 

Figure	Exp. Condition	Dunnett *p*-value	Dunn *p*-value	Unpaired t-test
Figure 4a	ECR65 Deletion 3		0.0011	
	ECR65 Deletion 4	< 0.0001		
	ECR65 Deletion 5	< 0.0001		
Figure 4c	ECR9 Deletion F			0.0016
	ECR9 Deletion 1			0.006
	ECR9 Deletion 2			0.0025
Figure 4b	ECR65 bHLH Mut A	< 0.0001		
	ECR65 hb Mut	0.0242		
Figure 4d	ECR9 site 3 Mut	0.0007		
	ECR9 site 4 Mut	0.001		
Figure 5a	ECR65 + Nhlh1	0.046		
	ECR9 + NeuroD1	0.0019		
	ECR9 + NeuroG2	0.0085		
	ECR9 + NeuroD4	0.021		
Figure 5c	ECR65 + Nhlh1	0.046		
Figure 5d	ECR9 + NeuroD1	0.0137		
	ECR9 + NeuroD4	0.01		
	ECR9 + NeuroG2	0.0164		
Figure 7a	Nhlh1 Vsx2 + GFP			0.035
	ECR9 + NeuroD1 Otx2	0.0245		
	NeuroD1 Vsx2 + GFP	0.0287		
	ECR9 + NeuroG2 Otx2	0.0098		
	ECR9 + NeuroD4 Otx2	0.0069		
	NeuroD4 Vsx2 + GFP	0.037		

## Results

### Regulatory element identification

**Fig. 1:**
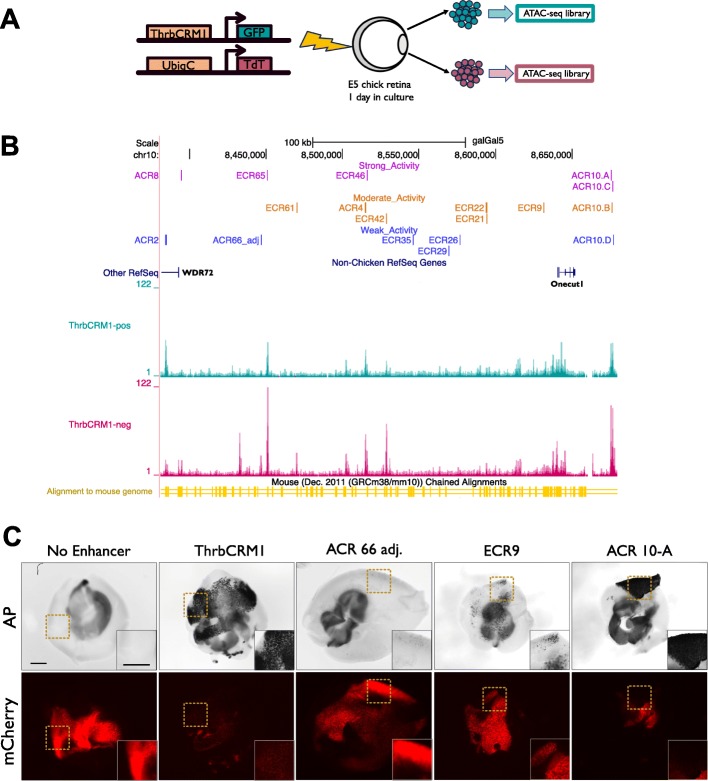
Identification and initial screening for regulatory elements active in E5 chick retinae. (**a**) Generation of ATAC-seq libraries of ThrbCRM1-positive and ThrbCRM1-negative cell populations. Chick retinae at embryonic day 5 (E5) were electroporated ex vivo with both ThrbCRM1::GFP and UbiqC::TdT plasmids and incubated in culture for 18–22 h prior to dissociation. Dissociated cells were sorted via FACS into GFP and TdT double-positive cells, and GFP-negative, TdT-positive cells. Each population was processed for ATAC-seq. (**b**) Visualization of aligned ATAC-seq reads to the galGal5 genome in UCSC Genome Browser in intergenic region between Onecut1 and WDR72 (labelled). Active regulatory elements represented by colored, labelled lines based on activity level from alkaline phosphatase assay (**c**) Alkaline phosphatase reporter assay to screen for regulatory activity. E5 chick retinae were electroporated with CRE::AP plasmids and CAG::mCherry plasmids. Empty Stagia3 vector (No Enhancer) represents the negative control. Dotted boxed region corresponds to magnified inset shown in bottom right corner. Scale bars in the first panel and inset represent 500 μm and apply to all panels

Two methods were employed to identify candidate cis-regulatory elements for Onecut1 (Fig. 1a,b). We first examined the intergenic region 5′ of the chicken Onecut1 coding region and 3′ of WDR72, as well as a short stretch of the intergenic region 3′ of the Onecut1 coding region for evolutionarily conserved regions (ECRs). As not all cis-regulatory sequences are strongly conserved and as ECRBrowser [32] utilizes an older chick genome assembly, we also used chromatin accessibility as a means of candidate enhancer identification. Chick E5 retinae were electroporated with ThrbCRM1::GFP and UbiqC::TdT, cultured for 18–22 h ex vivo and sorted into two populations: ThrbCRM1(+) cells, which were marked by both reporters and ThrbCRM1(−), which were marked by only TdT. These cells were then processed for ATAC-seq [7], aligned against the galGal5 assembly and the data was visualized in the UCSC Genome Browser [21] to identify accessible chromatin regions (ACRs) as potential cis-regulatory elements. These ATAC-seq libraries enabled the identification of chromatin regions with higher accessibility within the ThrbCRM1(+) population but many of the elements identified by conservation were accessible in both populations. Due to the possibility that enhancers may be in an “active” state in the ThrbCRM1(+) population as opposed an inactive but “primed” state [38] within the ThrbCRM1(−) population, these regions remained candidates. In total, we screened 98 ECRs and ACRs that were found in the Onecut1 region that encompassed the last intronic region of the WDR72 gene located upstream of Onecut1 and the Fam214a gene located downstream of Onecut1 (Additional File 1, Additional File 2) for their ability to drive reporter activity in the developing retina.

The first criterion of our screen was that the non-coding elements should be capable of driving reporter expression at E5 in the chick retina. To this end, we used a sensitive alkaline phosphatase (AP) reporter assay. Each potential cis-regulatory region was amplified from chick genomic DNA and cloned into Stagia3 [4], a dual GFP and AP reporter vector. Retinae at E5 (HH26) were electroporated with the reporter construct along with CAG::mCherry as the co-electroporation control. These retinae were cultured for approximately 18-22 h and then fixed before the AP stain was developed. AP reporter expression is visualized as a dark stain which can block out the fluorescence from the mCherry control in the case of very strong enhancers.

Previously identified Thrb reporters served as positive controls as they are known to be active in the E5 chick retina [11]. The empty Stagia3 vector served as the negative control to demonstrate the baseline levels of transcription when no cis-regulatory element is present. At this time point, the majority of the candidate sequences tested did not drive reporter expression above the baseline level defined by the negative control. The active elements that were initially chosen based on evolutionary conservation were largely found to have open chromatin states within the ATAC-Seq datasets. All active cis-regulatory elements (Fig. 1c, Additional File 3) were categorized as weak, moderate, or strong (Fig. 1b) based on the intensity of AP staining compared to the positive control as well as the overlap between the AP signal and mCherry.

### Regulatory activity within the cone/HC restricted RPC population

**Fig. 2:**
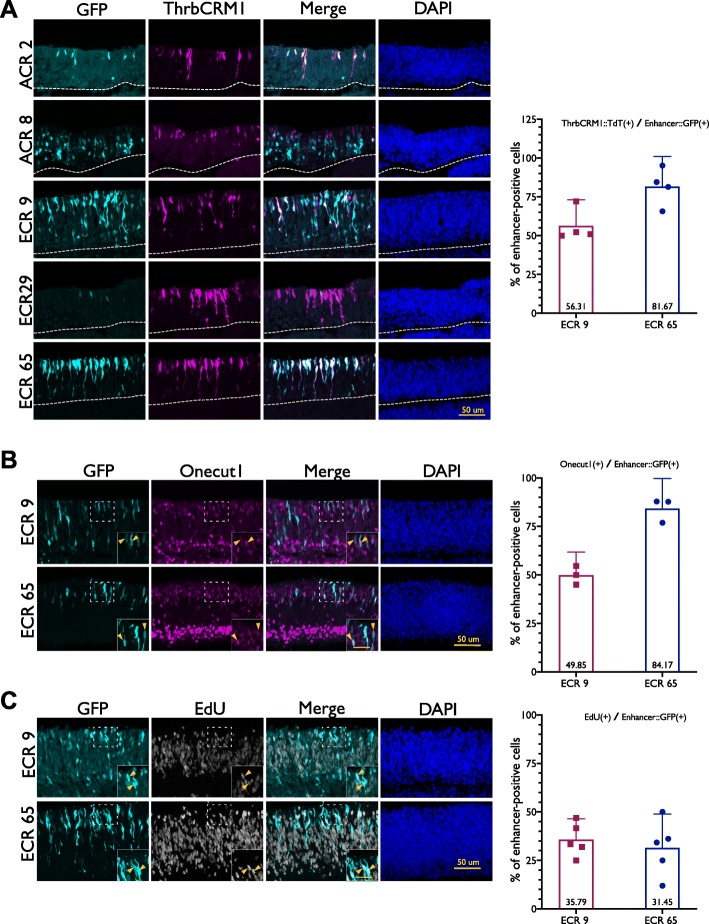
Candidate enhancers ECR9 and ECR65 demonstrate specificity to ThrbCRM1(+) population and overlap with mitotic progenitors. (**a**) Overlap between enhancer::GFP expression (cyan) and ThrbCRM1::AU1 expression (magenta) 18–22 h after electroporation. DAPI is shown in the last column. Electroporated retina is found above the dotted line. Quantification of ThrbCRM1 reporter(+) cells in ECR9::GFP and ECR65::GFP cells. Percentages of ThrbCRM1(+) cells were calculated from a flow cytometry assay in which retinae were electroporated with ECR9::GFP or ECR65::GFP and ThrbCRM1::TdT and the number of GFP/ TdT double-positive cells out of the total GFP(+) population was calculated. (**b**) Overlap of endogenous Onecut1 expression (magenta) with ECR9::GFP and ECR65::GFP (cyan) 18–22 h after electroporation. Yellow arrows indicate cells that are GFP(+) and Onecut1(+). Scale bar in inset represents 25 um and applies to all insets. Percentages of Onecut1(+) cells were calculated by determining the number of Onecut1(+) and GFP(+) double-positive cells out of all enhancer::GFP(+) cells. (**c**) Overlap of 1 h EdU-pulsed cells with ECR9::GFP and ECR65::GFP (cyan) 18–22 h after electroporation. Yellow arrows indicate cells that are GFP(+) and EdU(+). Scale bar in inset represents 25 um and applies to all insets. Percentages of EdU(+) cells were calculated from confocal images by determining the number of EdU and enhancer::GFP double-positive cells out of all enhancer::GFP-positive cells. Each point represents a biological replicate with data collected from two images. (**a**, **b**, **c**) Each point represents a biological replicate. Error bars represent 95% confidence interval

The second criterion of the screen was specificity to the population of fate-restricted early retinal RPCs that express Onecut1. The ThrbCRM1 element is active in the cone/HC restricted RPC population that expresses Onecut1 and Otx2 [11]. It has been previously reported that at 20 h post-electroporation, 30% of ThrbCRM1(+) cells are in S-phase or G2/M [6]. Therefore, to determine which active elements drove transcription in the same restricted RPC population, we co-electroporated the CRE::GFP reporter constructs with a ThrbCRM1::AU1 reporter into the chick retina at E5 and cultured overnight for approximately 20 h. Retinal sections were stained for AU1 and GFP and qualitatively evaluated with regards to the specificity of each active enhancer to the Onecut1(+) restricted RPC population marked by the AU1 reporter (Fig. 2, Additional File 4).

Many of the active CREs drove reporter expression in both the ThrbCRM1(+) and ThrbCRM1(−) populations, such as ECR42, ECR46 and ACR10-C (Additional File 4). ACR10-B (Additional File 4) activity appears to be distributed throughout the retina with no specific preference for ThrbCRM1(+) cells. Though the populations marked by these CREs overlap with our population of interest, they do not meet the criterion for specificity. Despite driving robust reporter expression in E5 chick retinae, ACR8 and ACR10-A (Fig. 2, Additional File 4) appear biased towards ThrbCRM1(−) cells. It is difficult to assess the specificity ECR26, ECR29, and ECR35 to the ThrbCRM1 RPC population due to the weak activity of these enhancers (Additional Files 3 and 4).

ACR2 does not exhibit activity in many cells, but most observed ACR2-positive cells are ThrbCRM1-positive (Fig. 2). ECR65 appears to be highly active in ThrbCRM1-positive cells and was qualitatively the most specific enhancer to the ThrbCRM1 population. Likewise, ECR9 activity appears biased to the ThrbCRM1(+) RPC population but drives reporter activity in some ThrbCRM1(−) cells.

This assay indicates that the active enhancers display a wide range in specificity to the ThrbCRM1(+) cell population (Fig. 2a, Additional File 4). ECR65, ECR9 and ACR2 are the most promising candidates for a regulatory element that contains the regulatory information sufficient to promote Onecut1 expression in RPC [CH]s. ECR9 and ECR65 were further assessed for specificity to this restricted RPC cell population as they demonstrated more robust reporter expression than ACR2.

To quantify the specificity of each enhancer to the Onecut1(+) restricted RPCs, E5 chick retinae were electroporated with ECR9:: or ECR65::GFP reporters, ThrbCRM1::AU1, and a co-electroporation control. It was observed that 56.31% of ECR9(+) cells and 81.67% of ECR65(+) cells were also marked by ThrbCRM1 (Fig. 2a). As further confirmation that Onecut1 is expressed in the populations marked by these two enhancers, we observed that 49.85% of ECR9(+) cells and 84.17% of ECR65(+) cells express Onecut1 (Fig. 2b). However, the ThrbCRM1(+) cell population does not consist of only RPCs. To determine the extent to which each enhancer was active in RPCs, E5 chick retinae electroporated with enhancer::GFP constructs were pulsed with EdU for one hour prior to harvest. Approximately 30% of ECR9(+) and ECR65(+) cells are marked by EdU (Fig. 2c), which is comparable to the proportion of EdU(+) cells within the ThrbCRM1 cell population [6]. Overall, these data provide evidence that the two regulatory elements ECR65 and ECR9 are active in ThrbCRM1 RPCs.

### History of ECR9 and ECR65 activity in early-born retinal cell types

To further test if these regulatory elements label RPCs which produce cells with the same fates that develop from the ThrbCRM1(+) RPC population, we used a PhiC31 lineage tracing system. In this system, a cis-regulatory element is used to drive the expression of PhiC31, which can activate a GFP responder vector through site-specific recombination to label cells with a history of cis-regulatory activity [36]. We combined this lineage trace system with immunohistochemistry and cell-specific markers to determine which cell types develop from RPCs marked by ECR65 and ECR9. For comparison, and to demonstrate that not every active regulatory element lineage traces to the same populations at this time point, we also lineage traced ACR2 and ECR42 (Additional File 5).

**Fig. 3:**
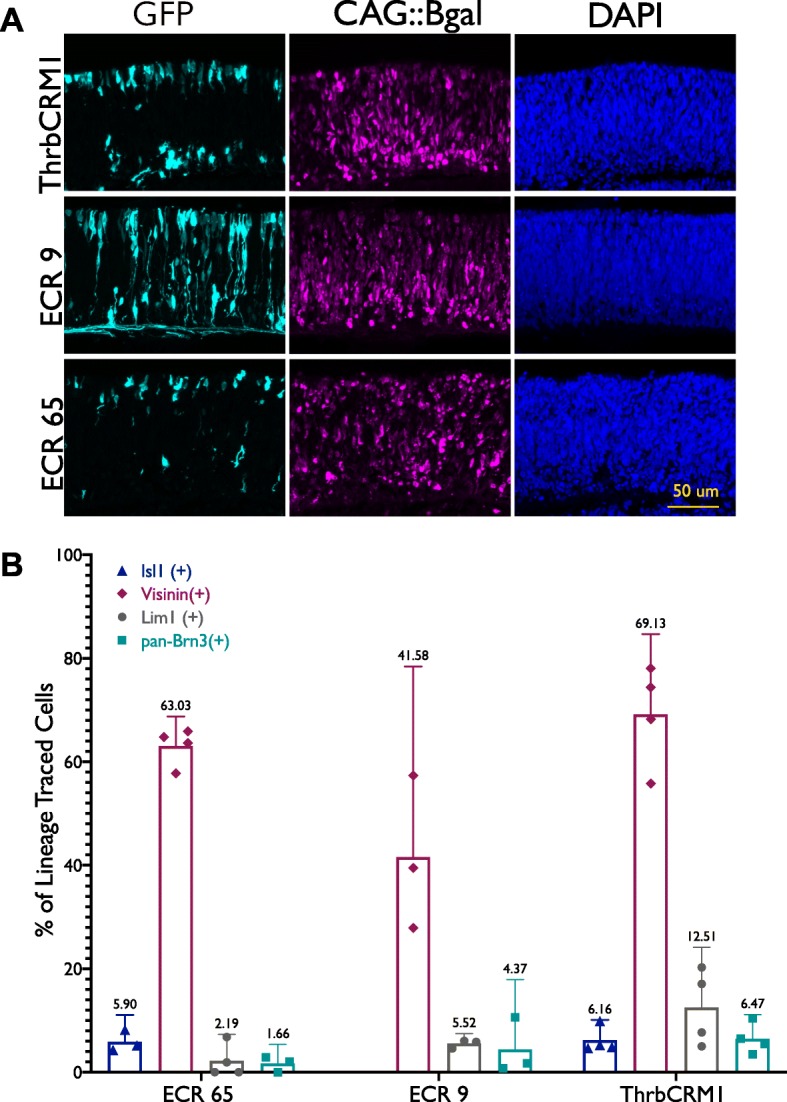
ECR65(+) and ECR9(+) cells lineage trace to similar cell fates as ThrbCRM1(+) population. E5 chick retinas were electroporated with enhancer::PhiC31 constructs and CAG::Bgal as an electroporation control before two days of tissue culture followed by harvest and immunohistochemistry. (**a**) Retinal sections were stained with GFP, Bgal, and DAPI. (**b**) Markers of early retinal cell types within lineage traced populations. Sections were stained with the markers Isl1, Visinin, Lim1, and pan-Brn3. Percentages of cells marked by these factors were calculated out of the total number of electroporated Bgal(+) cells per retinal section. Each point represents a biological replicate. Error bars represent 95% confidence interval

It was observed that approximately 40% and 5.5% of ECR9-lineage traced cells were cone photoreceptor (Visinin) and horizontal cell (Lim1) fates, respectively (Fig. 3b). However, not all ECR9-lineage traced cells correspond to one of these two cells types. The ECR9 lineage includes long axonal projections which appear to originate from GFP-positive cells in the innermost retina, suggesting that ECR9 is active at some point in the formation of retinal ganglion cells (RGCs). 4.37% of all GFP(+) cells were positive for pan-Brn3 (Fig. 3b), suggesting the presence of RGCs arising from ECR9(+) cells. This may indicate that ECR9 participates in the gene regulatory network responsible for generating cones, HCs, and RGCs. It is worth noting that the ThrbCRM1 lineage-trace also includes a similar percentage of pan-Brn3(+) cells as ECR9, despite the lack of inner retinal projections seen with ThrbCRM1 and previous *in ovo* lineage tracing of ThrbCRM1 that detected only a small number of RGCs [36]. This is potentially due to the antibody’s specificity – it may not mark all Brn3(+) RGC populations or it may be marking cells outside of the target population, including other inner retinal cells such as horizontal or amacrine cells.

Under the same experimental conditions, ECR65::PhiC31 marked overall fewer cells. However, the cell types with a history of ECR65 appeared biased towards the outer retina (Fig. 3a). 63.3% of GFP-positive cells were also marked by Visinin (Fig. 3b), confirming that ECR65-positive RPCs are capable of giving rise to cone photoreceptors. Surprisingly, very few ECR65-positive cells are marked by Lim1 despite the clear presence of GFP in inner retinal cells (Fig. 3a) and reported data that the ThrbCRM1 population gives rise preferentially to Lim1-positive HCs [36]. To determine whether these cells may be RGCs or Isl1-positive HC, we stained with pan-Brn3 and Isl1. There were few pan-Brn3(+) GFP(+) cells. However, 5.9% of all GFP-positive cells were marked by Isl1. Lineage tracing of ThrbCRM1 yields similar results, with 6.16% of GFP(+) cells also positive for Isl1 (Fig. 3b). In addition to RGCs and HCs, Isl1 has been reported to mark cholinergic amacrine cells (ACs) in the chick retina [10]. Though the ECR65 lineage is expected to be similar to that of ThrbCRM1, which does not include ACs, we cannot rule out that some of the Isl1+ cells in the ECR65 lineage are ACs [36].

ACR2 does not lineage trace to very many cells, but is nearly exclusive to the outer retinal cells marked by Visinin, indicating that ACR2’s role in retinal development is specific to photoreceptors, which are predicted to be cones at this timepoint. The lineage tracing of ECR42 illustrates that this enhancer’s activity is not limited to the cone and horizontal cell fate and marks a much broader RPC population, as evidenced by the pan-retinal distribution of cells with a history of this regulatory element (Additional File 5).

At the conclusion of our screen, eighteen active regulatory elements were identified in the retina, two of which drove GFP reporter expression in a spatial pattern that excluded ThrbCRM1(+) cells. ACR10-B was found to drive expression non-specifically throughout the retina and three elements, ECR26; ECR 29; and ECR35, drove expression too weakly to determine their specificity. ECR22 was found to mark a population that excludes cone photoreceptors (Gonzalez and Schick et al., in preparation). ACR2, ECR9, and ECR65 best met the criteria for our screen. These elements are either specific or strongly biased to the ThrbCRM1(+) population, mark early retinal RPC cells expressing Onecut1, and are active in cells that give rise to three early retinal fates: cone photoreceptors; horizontal cells; and RGCs. As the lineage marked by ACR2 is largely specific only to a smaller population of cone photoreceptors, the remainder of this study is focused on evaluation of ECR9 and ECR65.

### Bioinformatic analysis of regulatory element sequences

Though reporter assays indicate that ECR65 and ECR9 drive transcription in the ThrbCRM1(+) population during the cone and HC specification windows in the chick retina, we do not know what role these regulatory elements play in the GRN that gives rise to cone photoreceptors and horizontal cells.

To determine which transcription factors bind to ECR65 and ECR9, we first attempted to identify conserved motifs present within the sequence. We used UCSC Blat [20] to find homologous sequences to the originally identified chick ECR65 sequence from the golden eagle, barn owl, American alligator, thirteen-lined ground squirrel, northern treeshrew, chimpanzee, and human. ECR65 is well-conserved among all of the avian species as well as the American alligator and is conserved between avians and mammals.

When searching for the 515 bp chick sequence in the mouse genome, UCSC BLAT returned a 214 bp homologous stretch (Additional File 6A). This 214 bp mouse sequence contained a 58 bp stretch that did not align to the chick sequence. In contrast with chick ECR65, which is wholly located within accessible chromatin, only 150 bp of the homologous mouse sequence are in accessible chromatin region. The accessible chromatin extends past the homologous sequence. Mouse ECR65 (mECR65), as defined by chromatin accessibility, is 295 bp long (Additional File 6A). In summary, ECR65 demonstrated both a large sequence divergence and an apparent shift in chromatin accessibility between avian and mammalian species.

**Fig. 4:**
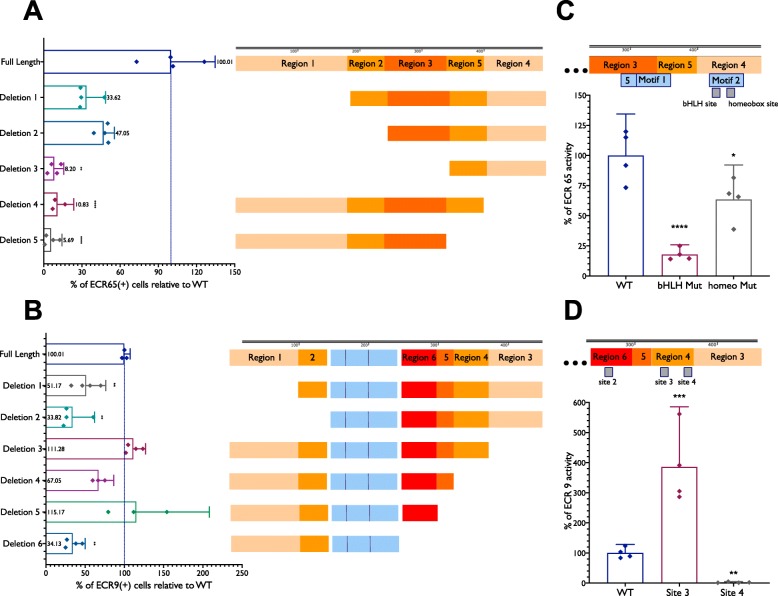
Deletions and mutations reveal sites important for regulatory activity. E5 chick retinae were electroporated with full length (**a**) ECR65 or (**b**) ECR9 driving TdT along truncated or mutated versions of the enhancers and CAG::IRFP as a co-electroporation control. Retinae were cultured for 18–22 h before dissociation and analysis by flow cytometry. Labelled blocks in (**a**) and (**b**) represent the enhancer constructs labelled along the Y-axis. Grey blocks in (**c**) and (**d**) denote putative TF binding sites. Deleted versions of enhancers were oriented in the expression vector such that the truncated end is farther from the TATA box. Percent of enhancer activity was calculated as a ratio between the total GFP(+) cells and the total TdT(+) cells and scaled to the activity of the full-length enhancer. Error bars represent 95% confidence interval. See Methods section for statistical tests

To assay this 295 bp mECR65 element for activity, we cloned it into the Stagia3 vector. When mECR65::GFP was electroporated into the chick retina at E5 along with ThrbCRM1::AU1, mECR65(+) cells were observed in the ThrbCRM1 population, which suggests that despite the sequence divergence, mECR65 has retained the regulatory information for activity in these cells. MEME motif analysis [3] of the various species-specific ECR65 sequences revealed that three motifs appeared conserved between avian and mammalian species (Additional File 6B). It is therefore likely the TF binding sites important for ECR65 activity are within the conserved Motifs 1, 5, and 2 (Fig. 4).

The ECR9 sequence is within accessible chromatin in both chick and mouse early retinal cells. The ECR9 sequence used was cloned out of the mouse genome and as seen above, is able to drive transcription of a reporter in chick retinal cells during the peak of cone and horizontal cell development. Though the sequence tested is over 400 bp long, only a ~ 270 bp span is conserved between mouse and chick. Motif analysis of this enhancer through MEME returns seven motifs, of which six are conserved between birds and mammals (Fig. 4, Additional Files 6C and 7).

### Deletions and mutations of regulatory element sequences

To take an unbiased approach to determine which regions of ECR65 are functional, five serial deletions of the chick ECR65 sequence were tested for their ability to drive reporter activity (Fig. 4, Additional File 7). The full-length/wild type version of ECR65 driving TdTomato was compared against the truncated versions driving GFP using flow cytometry. To ensure that any observed effects were not due to changes in the proximity between TF binding sites and the TATA box, all deletions were oriented such that the distance of all remaining sequence to the TATA box was preserved and these deletion constructs were compared to the full-length enhancer in the same orientation. To determine the effect of each deletion, we calculated the percent of GFP(+) cells relative to the amount of TdT(+) cells. WT ECR65::GFP marks about 56–65% as many cells as WT ECR65::TdT (Additional File 8). When these control values were normalized to 100%, ECR65 Deletions 1–3 respectively marked 33.62%, 42.05% and 8.2% as many cells as the control WT ECR65::GFP (Fig. 4a). When deletions were made on the other end of the regulatory element, GFP driven by ECR65 Deletion 4 or Deletion 5 respectively marks 10.82% and 5.68% as many cells as WT ECR65. Region 4 also encompasses MEME-predicted Motif 2. The severe loss of GFP expression upon deleting the 80 bp Region 4 suggests that Region 4 of ECR65 contributes significantly to the activity of this regulatory element.

ECR65 Motif 2 within Region 4 contains a potential binding site for a bHLH transcription factor. bHLH binding sites, known as E-boxes, typically follow the sequence CANNTG. Mutation of this potential E-box sequence resulted in a significant loss of enhancer activity (Fig. 4c, Additional Files 7 and 8). This result suggests that the functional sequence within Region 4 may be this 6-bp motif, predicted by TOMTOM [15] to bind the transcription factor NHLH1/NSCL1. Another mutation within Region 4 encompassing a predicted homeobox TF binding site resulted in some loss of GFP reporter activity (Fig. 4c, Additional File 8). The identification of these predicted TF binding sites and the resulting loss of transcriptional output does not rule out the presence of other functionally important binding sites in ECR65 Regions 1–3, particularly considering the substantial loss of activity from Deletion 3.

A similar deletion strategy was used to investigate ECR9. First, deletion constructs were tested in which the ECR9 sequences on either side of the MEME-identified motifs, labelled as Region 1 and Region 3, were removed. Additional deletion constructs removed four of the identified motifs, labelled as Regions 2, 4, 5, and 6 (Fig. 4, Additional File 7). As a control, we calculated the percent of GFP(+) cells relative to the amount of TdT(+) cells marked by full length versions of ECR9 (Additional File 8). Once again, we ensured that deletions were orientated away from the TATA box and compared only to full-length ECR9 of the same orientation. Deletions of Region 3, Region 4, and Region 5 did not result in a significant change to ECR9 activity. However, deletions of Region 1, Region 2, and Region 6 all resulted in a decrease of ECR9 activity as compared to the full-length enhancer. Examination of the sequence for putative TF binding sites led to four predicted bHLH sites (Fig. 4, Additional File 7). Site 1 and Site 2 are located in Region 1 and Region 6, respectively. Sites 3 and 4 are both located within Region 4. We hypothesized that if any of these sites were important for ECR9 function, mutation of one or more of them directly would result in a change in reporter expression. Mutating Site 3, located within Region 4, resulted in nearly a 300% increase in ECR9 activity whereas mutating Site 4 within the same region resulted in a 97.5% loss of ECR9 activity (Fig. 4d, Additional Files 7 and 8). The opposing effects observed from mutating these two sites indicate that while Site 4 normally functions as a transcriptional activator site, it is likely that Site 3 functions as a repressor. The lack of effect from ECR9 Deletion 4 may then be due to the loss of both of an activator and a repressor that function together to generate the correct transcriptional output from ECR9. However, it is also indicative that the remaining bHLH sites within ECR9 may be able to compensate for the loss of both Site 3 and Site 4. The loss of reporter output from Deletion 6, in which bHLH Site 2, Site 3, and Site 4 are all deleted, suggests that Site 2 is another important activator site.

### Interactions with bHLH transcription factors

Our screen for cis-regulatory elements that could regulate Onecut1 expression in early restricted RPCs led to ECR9 and ECR65, which appear to be active in overlapping populations of early RPCs that give rise to distinct subsets of retinal cell types. The tested serial deletions and mutations suggest that both of these elements have critical regulatory input from bHLH family transcription factors. In addition to the bioinformatically predicted Nhlh1 binding site in ECR65, bulk RNA-seq indicated that NeuroD1, NeuroD4, NeuroG2, and Atoh7 transcripts were enriched in the ThrbCRM1(+) population [6] and therefore also candidates to interact with these two cis-regulatory elements. To explore these possibilities, each of the five bHLH factors was overexpressed under the control of the ubiquitous CAG promoter in the E5 chick retina along with ECR65 and ECR9.

**Fig. 5:**
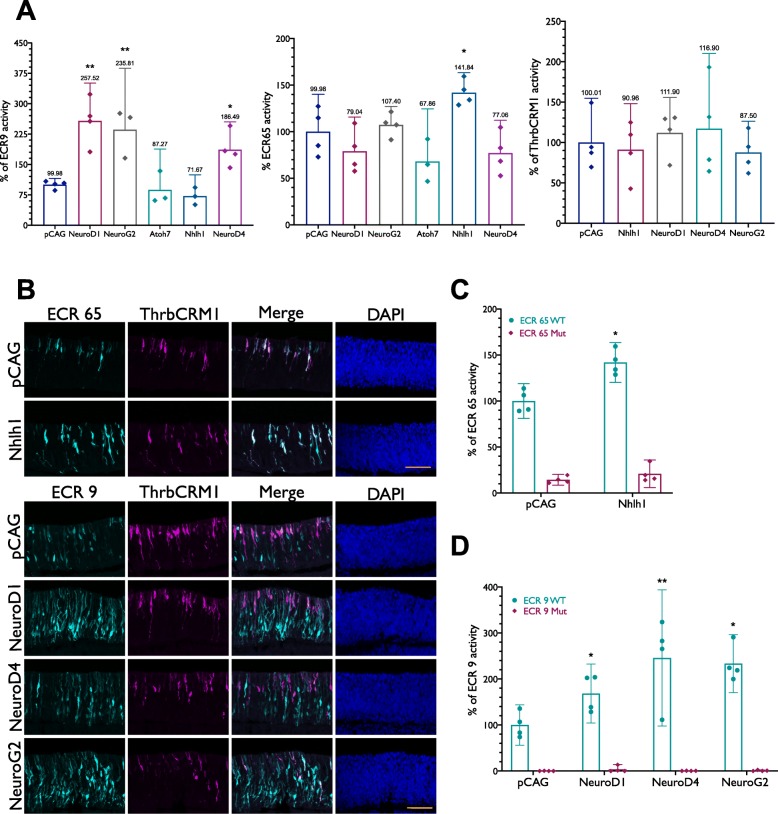
Effect of overexpression of bHLH factors on regulatory activity of ECR9, ECR65, and ThrbCRM1. (**a**) E5 retinae were electroporated with Nhlh1, NeuroD1, NeuroD4, NeuroG2 or Atoh7 under the control of the CAG promoter in combination with ECR65, ECR9 or ThrbCRM1 driving either TdT or GFP. Percentages of ECR9(+), ECR65(+) and ThrbCRM1(+) cells were calculated out of the total cells marked by co-electroporation control CAG::IRFP. (**b**) Retinae were electroporated with ECR9::GFP or ECR65::GFP in combination with ThrbCRM1::AU1 and a CAG::bHLH plasmid. (**c**, **d**) Comparison of the effect of bHLH overexpression on mutant and WT versions of ECR9 and ECR65. Percentages were calculated out of total number of CAG::IRFP(+) cells. Percentages of WT enhancer(+) cells in the control condition were scaled to 100% for (**c**, **d**). Percent of enhancer(+) cells in the empty CAG vector control condition were normalized to 100% for **a**. Error bars represent 95% confidence interval. In all graphs, each datapoint represents a biological replicate. See Methods for statistical tests

Under control conditions in which CAG did not drive any open reading frame, we calculated the percent of ECR9(+) or ECR65(+) cells out of the total electroporated population. Overexpression of either NeuroD1, NeuroG2, and NeuroD4 induced an increase in ECR9 reporter output, while Nhlh1 and Atoh7 did not. Confocal microscopy showed that the increase in ECR9(+) cells upon overexpression NeuroD1, NeuroG2, and NeuroD4 was predominantly in the inner retina (Fig. 5b). However, individual overexpression of these three bHLH factors is not sufficient to increase activity of the ECR9 Site 4 mutation (Fig. 5d).

We detected no change to the ECR65 reporter output upon misexpression of NeuroD1, NeuroD4, NeuroG2, or Atoh7 (Fig. 5a). However, overexpression of Nhlh1 led to a significant increase in the number of GFP(+) cells. Confocal microscopy data showed that the population marked by ECR65 appears to expand towards the inner retina upon Nhlh1 overexpression, with the morphology of some cells resembling multipotent RPCs marked by Vsx2 (Fig. 5a, b). Furthermore, Nhlh1 overexpression was unable to rescue the loss of GFP activity seen in ECR65bHLH Mut1::GFP, suggesting that the site CATCAG within ECR65 is not only required for regulatory activity, but possibly mediates the interaction between ECR65 and Nhlh1 (Fig. 5c).

Despite their effects on ECR9 and ECR65 activity, none of the four candidate bHLH factors were able to increase GFP reporter output driven by ThrbCRM1 (Fig. 5a) or drive any changes in the spatial activity of the enhancer in the chick retina at E5 (Fig. 5b). In addition, none of the bHLH genes were sufficient to ectopically induce ThrbCRM1 activity in the P0 mouse retina, suggesting that these genes were not sufficient to induce Onecut1 expression at this time (Additional File 9). These results suggest that either ECR9 and ECR65 activation may occur after ThrbCRM1 activation or that the interactions between the bHLH factors and enhancers are not sufficient to induce Onecut1 expression.

### Timeline of regulatory element activity

**Fig. 6:**
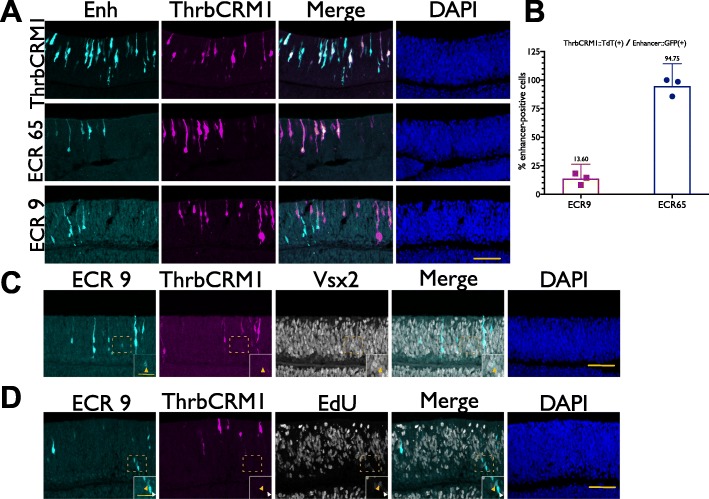
Onset of ECR9 and ECR65 activity compared to ThrbCRM1 (**a**) E5 retinae were electroporated with ThrbCRM1::TdT and either ThrbCRM1::GFP, ECR65::GFP or ECR9::GFP and cultured for 8 h before harvest. Immunohistochemistry was used to amplify GFP and TdT signal. (**b**) Quantification of ThrbCRM1(+) cells within ECR9(+) population and ECR65(+) population. Each data point represents a biological replicate. Error bars represent 95% confidence interval (**c**) E5 retinae were electroporated as in (**a**) but also stained to detect Vsx2. Yellow arrow indicates a cell that is ThrbCRM1::TdT(−), ECR9::GFP(+), Vsx2(+). (**d**) E5 retinae electroporated with ECR9::GFP and ThrbCRM1::TdT were cultured for 8 h and pulsed with EdU from hour 7–8. Yellow arrow indicates a cell that is ECR9::GFP(+) ThrbCRM1::TdT(−) EdU(+). White arrow indicates a cell that is ECR9::GFP(+) ThrbCRM1::TdT(−) EdU(−). Scale bars in (**a**, **c**, **d**) represent 50 μm and apply to all panels. Scale bars in (**c**) and (**d**) insets represent 20 μm and apply to all panels

ECR65::TdT and ECR9::GFP were electroporated with ThrbCRM1::GFP or ThrbCRM1::TdT respectively into retinas that were then cultured for 8 h to assess whether the two regulatory elements activated at the same time as ThrbCRM1. ECR65, ECR9, and ThrbCRM1 are all able to drive reporter expression 8 h after electroporation. At this early time point, similar to the 18–22 h time point, almost all ECR65(+) cells are ThrbCRM1(+) (Fig. 6a, b). At 8 h, the ECR9(+) population contains both ThrbCRM1(+) and ThrbCRM1(−) (Fig. 6a). It is striking that at the 8 h timepoint, the more intensely ECR9::GFP(+) cells appear to be ThrbCRM1(−) (Fig. 6a). Only 13.6% of ECR9(+) cells are also ThrbCRM1(+) at 8 h (Fig. 6b), compared to 56.31% at 18–22 h. These results indicate that onset of ECR9 and ECR65 activity is not later than or dependent on ThrbCRM1 activation. Upon further examination of the ECR9(+) ThrbCRM1(−) population, it was observed that some of these cells are progenitors that can be labelled with EdU (Fig. 6c). However, very few of them were observed to be Vsx2(+). This data suggests that ECR9 is active in ThrbCRM1(−) cells before it is active in ThrbCRM1(+) cells and that there exists a progenitor population which is ECR9(+) ThrbCRM1(−) Vsx2(−).

### Molecular events upstream of ThrbCRM1 activity

**Fig. 7:**
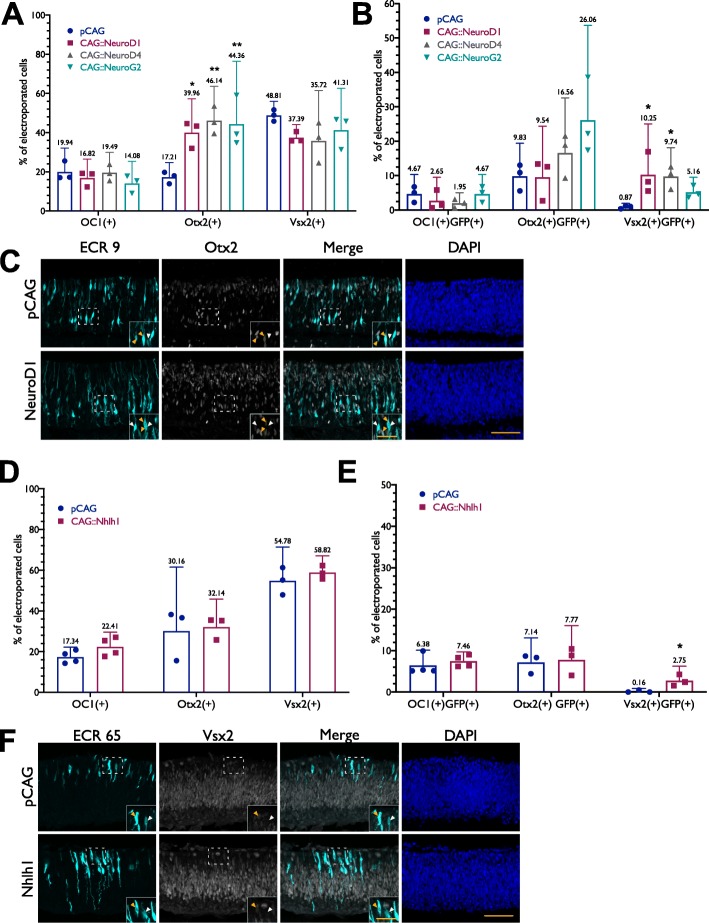
Co-localization of bHLH-induced activity of ECR9 and ECR65 with early retinal development markers. E5 retinas were electroporated with ECR9::GFP or ECR65::GFP and their corresponding bHLH factors and cultured for 18–22 h before harvest and immunohistochemistry. (**a**) Cell quantitation derived from confocal images of retinas stained for co-electroporation marker Bgal, and either Vsx2, Otx2 or OC1. Percentages of Otx2(+), Vsx2(+), or OC1(+) cells were calculated out of the total number of Bgal(+) cells. (**b**) Cell quantitation derived from confocal images of retinas stained for ECR9::GFP, co-electroporation marker Bgal, and either Vsx2, Otx2 or OC1. Percentages of ECR9(+) Otx2(+), ECR9(+) Vsx2(+), or ECR9(+) OC1(+) cells were calculated out of the total number of Bgal(+) cells. Error bars represent 95% CI (**c**) Confocal images of retinas stained for ECR9::GFP and Otx2. Yellow arrows denote ECR9(+) Otx2(+) cells. White arrows denote ECR9(+) Otx2(−) cells. (**d**, **e**) Same as for (**a**, **b**) but for ECR65::GFP. **f** Confocal images of retinas were stained for ECR65::GFP and Vsx2. Yellow arrows depict ECR65(+) Vsx2(+) cells. White arrows depict ECR65(+) Vsx2(−) cells. Scale bar in (**c,f**) represents 50 μm and applies to all panels. Scale bar in insets represents 25 um and applies to all panels

We then sought to determine whether any of the bHLH factors which impact ECR9 and ECR65 activity affected one or more of the factors upstream of ThrbCRM1 such as Onecut1 and Otx2, or affected expression of the multipotent gene Vsx2. Overexpression of NeuroD1, NeuroD4, or NeuroG2 individually resulted in an increase of electroporated Otx2(+) cells (Fig. 7a). Together with the increase in ECR9::GFP(+) cells, this would suggest that the newly GFP(+) cells are expressing Otx2. As there is no change in the proportion of GFP(+) Otx2(+) cells upon overexpression of each bHLH factor (Fig. 7b) and many of the newly GFP(+) cells in the inner retina are Otx2(+) (Fig. 7c, yellow arrows), it is also important to note that this result suggests that ECR9 is not turning on in previously ECR9(−) Otx2(+) cells. We then examined whether this enhancer-marked population shared a relationship with the Vsx2(+) multipotent RPC population. Vsx2, shown to be largely absent in the ThrbCRM1(+) restricted RPC population, marks 48.8% of all electroporated cells after one day in culture while ECR9(+) Vsx2(+) cells comprise less than 1% of all electroporated cells (Fig. 7a, b). Overexpression of bHLH factors led to an increase in ECR9(+)Vsx2(+) cells (Fig. 7b), suggesting that Vsx2(+) cells are now being recruited to the ECR9(+) population. There was a trend for Vsx2(+) cells within the electroporated population to decrease upon bHLH overexpression but this was not statistically significant (Fig. 7a). None of the bHLH factors associated with ECR9 were able to induce Onecut1 expression in the electroporated population (Fig. 7a) and no change in the percent of ECR9(+) Onecut1(+) cells was observed (Fig. 7b). These results indicate that the some of the ECR9(+) newly inner retinal cells can be marked by Otx2 but that this population is largely Onecut1(−) and Vsx2(−).

Overexpression of Nhlh1 does not result in any change to electroporated Otx2(+) (Fig. 7d) cells nor to those cells positive for both Otx2 and ECR65::GFP (Fig. 7e). Staining with Vsx2 confirmed that there is an increase in ECR65(+) Vsx2(+) cells upon overexpression of Nhlh1 (Fig. 7e, f), as suggested by the data from Fig. 5, though the overall percent of Vsx2(+) cells out of the total electroporated population does not change (Fig. 7d). This result suggests that Nhlh1 overexpression induces some ECR65 activity within the Vsx2(+) cell population. Like the Otx2(+) cell population, the Onecut1(+) and Onecut1(+) ECR65(+) cell populations remain unaffected by Nhlh1 overexpression.

### Relationship between bHLH factors and early-born retinal cell types

We investigated the possibility of these factors affecting retinal cell types developing from E5 to E7. Previously it has been shown that overexpression of Onecut1 in the postnatal mouse retina can induce an increase in cones and horizontal cells while suppressing the rod photoreceptor fate [11]. Here, we overexpressed the four candidate bHLH factors, cultured for two days to mirror the lineage tracing experiments, and stained for the same cell-type specific markers (Additional File 10) to determine whether these bHLHs play a role in the development of the cell types marked by the lineage tracing of ECR65 and ECR9.The lineage tracing of ECR9 in conjunction with the data from overexpression of NeuroD1, NeuroD4, and NeuroG2 suggests that one or more of these transcription factors may play a role in the development of Lim1(+) horizontal cells or RGCs. However, no conclusive increase of these cell types was observed upon bHLH overexpression. Consistent with previously reported data [24] overexpression of Nhlh1 was not sufficient to induce an increase in Isl1(+) cells. Lastly, none of the four candidate bHLH factors had an effect on photoreceptors marked by Visinin.

## Discussion

The work presented here is intended to serve as a step towards understanding the distinction between cell fate multipotency and restriction. The expression of multipotent RPC genes such as Vsx2 and genes such as Onecut1 that mark the ThrbCRM1(+) fate-restricted RPC population appear to be mutually exclusive [6]. It is not understood what gene regulatory networks are involved in the establishment of these two populations. We therefore sought to identify cis-regulatory elements specific to the RPC [CH] population that function upstream of Onecut1 and may be involved in the restriction process.

Our large-scale, multi-step screen for regulatory elements resulted in the identification of three regulatory elements that drove a spatial expression pattern biased to the cone/HC restricted RPC population. In addition to ECR9 and ECR65, we found multiple regulatory elements which drove expression in non-specific expression patterns and two which completely excluded the population of interest. These two regulatory elements, ACR8 and ACR10-A, may be involved in generation or maintenance of the multipotent RPC population that gives rise to a broader range of mature cell types as well as the restricted RPC populations. It may also be that the more broadly-acting or ThrbCRM1(−) elements from this screen require further genomic context to act specifically in Onecut1(+) restricted RPCs. Our reporter assay is demonstrably effective in finding minimal elements that can drive specific spatiotemporal expression patterns, but we cannot be certain that we replicate the genomic function of every assayed element given the importance of chromatin state and the surrounding sequences. Our assay does capture some aspects of genomic context as every active element is found within accessible chromatin regardless of sequence conservation. Highly conserved elements located within closed chromatin in E5 chick retinal cells were not found to be active. Despite this, differential chromatin accessibility may not be a strong indicator of enhancer specificity in cell populations at the same developmental time point. ECR65 in particular is accessible in both ThrbCRM1(+) and ThrbCRM1(−) cells, yet under normal conditions is only active in the ThrbCRM1(+) population, demonstrating that appropriate combinations of transcription factors are still required to confer specific activity to regulatory elements.

The regulatory elements ECR9 and ECR65 mark distinct but overlapping populations of developing chick retinal cells from E5-E6, which includes RPCs (Fig. 2). ECR65 has also been identified through DNaseI hypersensitivity in the mouse retina as “OC1 A” [33] but has not been further characterized. Here, we report that the ECR65(+) population overlaps almost entirely with the ThrbCRM1(+) population of cone/HC RPCs (Fig. 2) and excludes the Vsx2(+) multipotent RPC population (Fig. 7). By combining bioinformatic analyses of the active enhancer sequences with functional tests and previously published RNA-seq data, we were able to connect the activity of ECR9 and ECR65 to bHLH transcription factors.

The overexpression assays demonstrate specificity in the interactions between the four candidate bHLH factors and the two candidate enhancers. The involvement of these transcription factors in retinal development and specifically in retinal cell fate choice has also been well-documented [8, 9, 17]. For example, Nhlh1 RNA is known to be present in Isl1(+) RGCs [24] and has been observed in scRNA-seq data from chick retinal cells to mark a cone photoreceptor subtype [13]. When overexpressed alongside both candidate enhancers, Nhlh1 is only able to affect the activity of ECR65 (Fig. 5a, b) which lineage traces to an Isl1(+) population (Fig. 3). However, further investigation is required to identify and characterize Nhlh1(+) developing cone photoreceptors and determine whether they develop from the ECR65(+) population. Similarly, NeuroG2 has been found to be important for RGC genesis [18, 30] and involved in HC fate choice [2]. This role may underlie its interaction with ECR9 (Fig. 5a, b), which lineage traces to horizontal cells and morphologically characteristic RGCs (Fig. 3).

The ECR9(+) population also distinguishes itself with a subpopulation that does not overlap with ThrbCRM1 activity. However, this ECR9(+) ThrbCRM1(−) population that includes RPCs also does not overlap strongly with the multipotent Vsx2(+) population (Figs. 6, 7). Though NeuroD1, NeuroG2, and NeuroD4 were considered candidate TFs largely due to their enrichment in the ThrbCRM1(+) population [6], our data did not indicate that overexpression of individual candidate bHLH factors affected ThrbCRM1 activity on the timescale that we examined. Both the quantitative and qualitative assessment along with the visible increase in both Otx2(+) and ECR9(+) cells in the inner retina suggest that NeuroD1, NeuroD4, and/or NeuroG2 mediate ECR9 activity in a population of Otx2(+) cells distinct from the ThrbCRM1(+) restricted RPC population. The requirement for sequence elements that match consensus bHLH binding sites suggest that there is direct regulation of ECR9 and ECR65 activity by bHLH factors. However, it is possible that the misexpression effects of the tested bHLH factors on ECR9 and ECR65 activity could be an indirect effect, mediated by expression changes in other bHLH factors or potentially other transcription factors that act through the sites defined in this study.

**Fig. 8:**
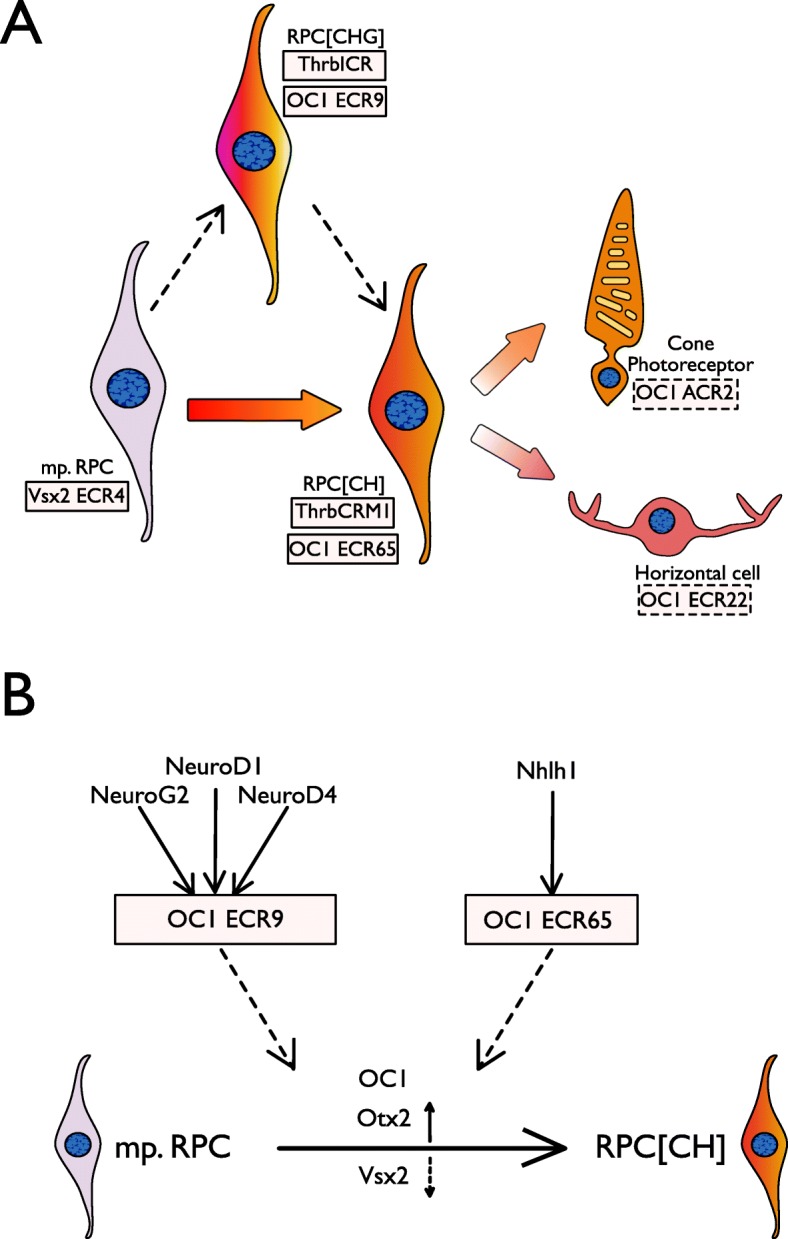
Model of ECR9 and ECR65 roles in cone/HC regulatory network. (**a**) Cell populations with ECR9 and ECR65 activity in relation to populations marked by previously published elements and OC1-associated elements reported here. Vsx2 ECR4 is active in the multipotent RPC population, whereas OC1 ECR9, OC1 ECR65, ThrbCRM1, and ThrbICR are all active in fate-restricted RPC populations. (**b**) Molecular events upstream and downstream of ECR9 and ECR65 activity. Multipotent RPCs give rise ultimately to RPC [CH] s, which corresponds to a down-regulation of Vsx2 and an upregulation of OC1 and Otx2. The bHLH factors that are sufficient to activate ECR9 and ECR65 reporter expression are shown

It has been hypothesized that an intermediate restricted RPC, marked by ThrbICR, gives rise to ThrbCRM1(+) restricted RPCs [36]. Lineage tracing of ThrbICR, which is bound by NeuroD1 [26], shows that the cells marked by that element can give rise to RGCs as well as cones and horizontal cells [36]. In conjunction with the ECR9 lineage tracing data and NeuroD1 overexpression data, ECR9 activity in cells which are neither ThrbCRM1(+) nor Vsx2(+) suggests that ECR9 could also label this intermediate population, referred to as RPCs [CHG] (Fig. 8a).

The regulatory elements uncovered in this and previous screens can therefore be used to mark distinct developing populations in the early vertebrate retina. OC1 ECR65 and ThrbCRM1 are both active in the restricted RPC [CH], while OC1 ECR9 and ThrbICR activity may be marking the hypothesized RPC [CHG] which is distinct from multipotent RPCs characterized by the activity of VSX2 ECR4 [6]. Other elements found in the screen, such as ACR2 (Fig. 2, Additional File 5) and ECR22 (Gonzalez and Schick et al., in preparation), may be more specific to either the cone photoreceptor precursors or horizontal cell precursors, respectively, from the ThrbCRM1 lineage (Fig. 8a). Though ECR9(+) and ECR65(+) cell populations overlap with each other and other CREs such as ThrbCRM1 and potentially ThrbICR, the two enhancers characterized here are unique in their responses to the bHLH factors described above. We hypothesize that the interactions between ECR9 and ECR65 and their respective bHLHs play a role in the transition from multipotent RPCs to the fate-restricted RPC [CH] population (Fig. 8b). It may be that one or more of the bHLH factors shown to interact with ECR9 may play a role in coordinating Vsx2 downregulation and activation of Onecut1 expression and ThrbCRM1 activity (Fig. 8b).

While we have not determined which factor(s) among NeuroD1, NeuroD4 and NeuroG2 interact with ECR9 in vivo, the ability of all three factors to increase ECR9 activity is consistent with a previous study demonstrating their functional redundancy during retinogenesis [2]. As ECR9 has multiple putative E-box sites, it may be that some combination of two or all three factors is required for the correct regulatory output of this enhancer. Of the ECR9 bHLH sites, Sites 2–4 all vary in sequence but all three sites and their flanking sequences are highly conserved between mouse, chick and human (Additional File 7). It is also worth noting that while the ECR65 bHLH site shares the same sequence as ECR9 Site 3, the two enhancers still differ in their ability to respond to Nhlh1 overexpression. This may be due to the differences in their flanking sequences. Previous work has shown that some bHLH factors are able to utilize each other’s binding sites [28] and that the sequence flanking the core E-box may also be important for binding affinity [14].

Our results do not suggest that any of these bHLH factors individually are capable of inducing or suppressing any of the early-born cell fates in the chick retina. Previous studies using Xenopus were able to yield an increase in photoreceptors upon overexpression of NeuroD1 and NeuroD4 [42]. In chick, NeuroD1 has been reported to induce more photoreceptors when virally overexpressed in the chick retina at E2 and cultured until E8.5/E9 [46]. Our lack of a similar result may be due to differences in the experimental timepoints, as our experimental conditions included a maximum culture time of 48 h. Though we were also not able to induce horizontal cell or RGC fates, it may be because the overexpressed factors require the co-expression of other TFs in order to specify or induce particular cell fates. For instance, the prediction of a homeobox TF binding site so close to the bHLH binding site in ECR65 may suggest that both Nhlh1 and a homeobox factor are required to induce the cell fates observed from lineage tracing ECR65. Future studies are required in which bHLH factors are overexpressed in combination with each other and with homeobox factors to determine which factors are sufficient to drive early retinal cell fates in chick.

## Conclusions

This study examined the upstream regulatory events of the Onecut1 gene that occur in chick RPCs. Guided by both sequence conservation and chromatin accessibility, we identified two regulatory elements near the Onecut1 gene, ECR9 and ECR65, that are preferentially active in Onecut1-expressing ThrbCRM1(+) RPCs. We find that both of these elements are predicted to contain bHLH transcription factor binding sites, which are required for activity of these elements. Overexpression of specific bHLH members leads to ectopic activity of ECR9 and ECR65 in Vsx2(+) RPCs. These bHLH factors are able to upregulate endogenous Otx2 expression, a protein normally expressed in fate-restricted RPCs. Taken together, these results suggest a role for bHLH factors in promoting the formation of fate-restricted RPCs from multipotent RPCs through the activation of Onecut1 and Otx2.

## Supplementary information

**Additional File 1.** Genomic map of potential regulatory elements identified near Onecut1 gene locus. Coding regions of Onecut1 and WDR72 from multiple species are marked by blue bars. The 5′ most transcriptional start site for Fam214a begins in the ACR10 region, as determined by examination of previous retinal transcriptome datasets [6], and Fam214a transcripts extend to the right. Yellow bars and lines at the bottom indicate sequence conservation between chick and mouse (mm10 assembly) genomes. Chromatin accessibility reads from the ThrbCRM1-positive population are shown in teal, reads from the ThrbCRM1-negative cell population are shown in magenta. Peaks in magenta may be cut off as the data was scaled to optimally visualize the ThrbCRM1 chromatin accessibility. Black bars at the top indicate potential regulatory elements, labelled as Evolutionary Conserved Region (ECR) if originally identified through sequence conservation and Accessible Chromatin Region (ACR) if identified through ATAC-seq data. Elements which could not be cloned or assayed for activity are marked with an asterisk.

**Additional File 2.** ID and genomic coordinates of all tested sequences in the galGal5 chick genome assembly, with the exception of ECR9 which is in the mm10 mouse genome assembly.

**Additional File 3.** Regulatory elements active in E5 chick retinae. E5 chick retinae electroporated with Enhancer::AP plasmids and CAG:: mCherry plasmids and cultured for 1 day prior to alkaline phosphatase assay. Shown are the AP reporter signal on top and the mCherry signal on bottom. Insets in AP panels show zoomed in areas of reporter activity. Scale bar in last panel represents 500 μm and applies to all.

**Additional File 4.** Overlap between ThrbCRM1 activity and activity of eleven candidate enhancers. E5 chick retinae were electroporated with enhancer::GFP (cyan) constructs as well as ThrbCRM1::AU1 (magenta) constructs and cultured for 18–22 h prior to antibody staining with GFP, AU1 and DAPI (nuclei) to determine which enhancers marked the same cell population as ThrbCRM1. Scale bar in last panel represents 50 μm and applies to all.

**Additional File 5.** Lineage tracing of regulatory elements reveals range in specificity. ACR2::PhiC31 and ECR42::PhiC31 were electroporated into E5 chick retinae with a PhiC31 GFP responder plasmid and CAG::Bgal and cultured for two days before harvest and staining with GFP to label cells with a history of PhiC31 expression and Bgal to label all electroporated cells. Scale bar in top right panel represents 50 μm and applies to all.

**Additional File 6.** Conservation of sequence, chromatin state and function of ECR65 and ECR9. (A) The entirety of chick ECR65 (purple bar) aligns to open chromatin in the chick genome. The homologous mouse sequence (grey bar with red lines) only partly aligns to the open chromatin region in the mouse. Mouse ECR65 (long black bar) is a longer region of open chromatin. Regions 2 and 6 (small labelled black bars) are conserved between both Mouse ECR65 and Chick ECR65. Insets show zoomed out genomic area to include surrounding closed chromatin. (B) Mouse ECR65::GFP was electroporated into E5 chick retina along with ThrbCRM1::AU1 and cultured for 18–22 h before harvest and immunohistochemistry. Retinae were stained for GFP, AU1 and DAPI to examine overlap between GFP and AU1. Scale bar shown in last panel represents 50 μm and applies to all. (C) Chromatin accessibility at the ECR9 region in the mouse E12.5 retina. The thick black bar depicts the mouse ECR9 region, the grey bars represent the regions of homology to the chicken, and the thin black bars represent motifs identified in the mouse ECR9 sequence.

**Additional File 7.** Sequence alignments of ECR9 and ECR65 mouse, chicken and human homologous sequences. Asterisks below nucleotides denote conservation. Labelled black arrows demarcate boundaries of Motifs or Regions that were deleted in Fig. 4. ECR65 Region 3 and ECR65 Region 5 share a boundary. All deletions are directional as shown in Fig. 4. Mutated bHLH sites are shown below full alignments, highlighted in blue.

**Additional File 8.** Unscaled values from deletion, mutation, and overexpression experiments (A) ECR65 activity from deletions and mutations corresponding to Fig. 4. SP52 and NJ849 refer to two different orientations of ECR65::GFP. (B) ECR65 activity with empty pCAG vector, corresponding to Fig. 5. (C) ECR9 activity from deletions and mutations, corresponding to Fig. 4. NJ1140 and NJ1142 refer to two different orientations of ECR9. (D) ECR9 activity with the empty pCAG vector, corresponding to Fig. 5. (E,F) Mutations of ECR65 and ECR9 with different mutant sequences, corresponding to Fig. 4. Error bars represent 95% confidence interval. Each depicted point represents a biological replicate.

**Additional File 9.** Candidate bHLH factors are not sufficient to induce ectopic ThrbCRM1 activity in the mouse postnatal retina. P0 mice retinae were electroporated with CAG::Bgal (magenta), ThrbCRM1::GFP (green) and the five candidate bHLH factors under the control of CAG. An empty CAG plasmid served as the negative control and CAG::OC1 as a positive control. Retinae were cultured for two days prior to harvest and staining with Bgal, GFP and DAPI.

**Additional File 10.** Individual bHLH factors are not sufficient to increase numbers of early retinal cell types. E5 chick retinae were electroporated with CAG::bHLH constructs and CAG::Bgal as an electroporation control and cultured for two days before being processed for immunohistochemistry. Retinal sections were stained with DAPI (nuclei), Bgal (electroporated cells) and cell-type specific markers. Percentages were calculated using the number of cells marked by each factor out of the total number of Bgal(+) cells. Error bars represent 95% confidence intervals. Each point represents a biological replicate.

## Data Availability

ATAC-seq data generated from chick retinal cells has been submitted to GEO as data set GSE141019 and will be available upon publication. ATAC-seq data generated from mouse retinal cells is not currently available as it is part of another study but can be made available upon request.

## Abbreviations

CRE: Cis-regulatory element
GFP: Green fluorescent protein
GRN: Gene regulatory network
HC: Horizontal cell
RPC [CH]: Restricted retinal progenitor cell that gives rise to cones, horizontal cells
RPC [CHG]: Restricted retinal progenitor cell that gives rise to cones, horizontal cells, retinal ganglion cells
RPC: Retinal progenitor cell
TdT: TdTomato
TF: Transcription factor
