# Supplementary figures and images for: Cis-regulatory analysis of Onecut1 expression in fate-restricted retinal progenitor cells
Source: Neural Dev. 2020 Mar 19;15:5. doi: 10.1186/s13064-020-00142-w (PMC7082998; doi:10.1186/s13064-020-00142-w)

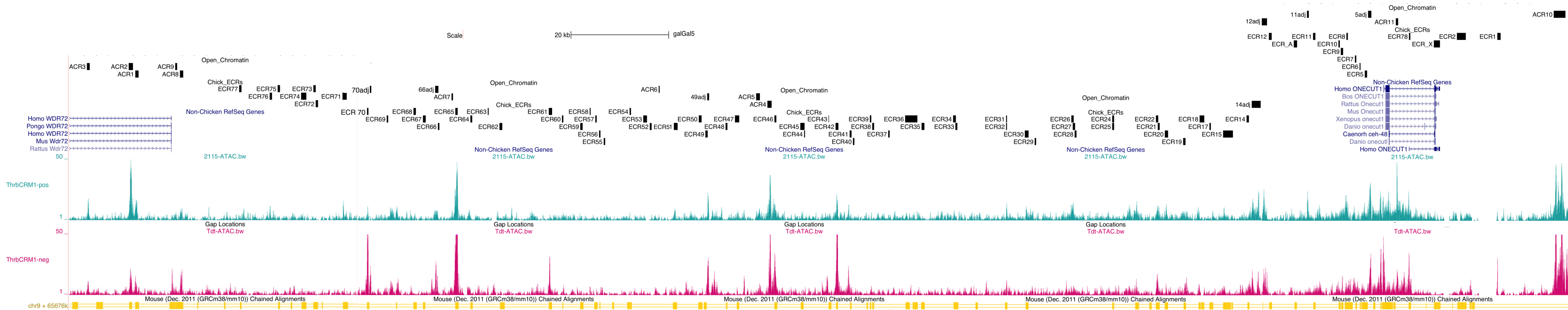

Supplement: Supplementary file 1 — Additional File 1. Genomic map of potential regulatory elements identified near Onecut1 gene locus. Coding regions of Onecut1 and WDR72 from multiple species are marked by blue bars. The 5′ most transcriptional start site for Fam214a begins in the ACR10 region, as determined by examination of previous retinal transcriptome datasets [6], and Fam214a transcripts extend to the right. Yellow bars and lines at the bottom indicate sequence conservation between chick and mouse (mm10 assembly) genomes. Chromatin accessibility reads from the ThrbCRM1-positive population are shown in teal, reads from the ThrbCRM1-negative cell population are shown in magenta. Peaks in magenta may be cut off as the data was scaled to optimally visualize the ThrbCRM1 chromatin accessibility. Black bars at the top indicate potential regulatory elements, labelled as Evolutionary Conserved Region (ECR) if originally identified through sequence conservation and Accessible Chromatin Region (ACR) if identified through ATAC-seq data. Elements which could not be cloned or assayed for activity are marked with an asterisk. [file 13064_2020_142_MOESM1_ESM.pdf]

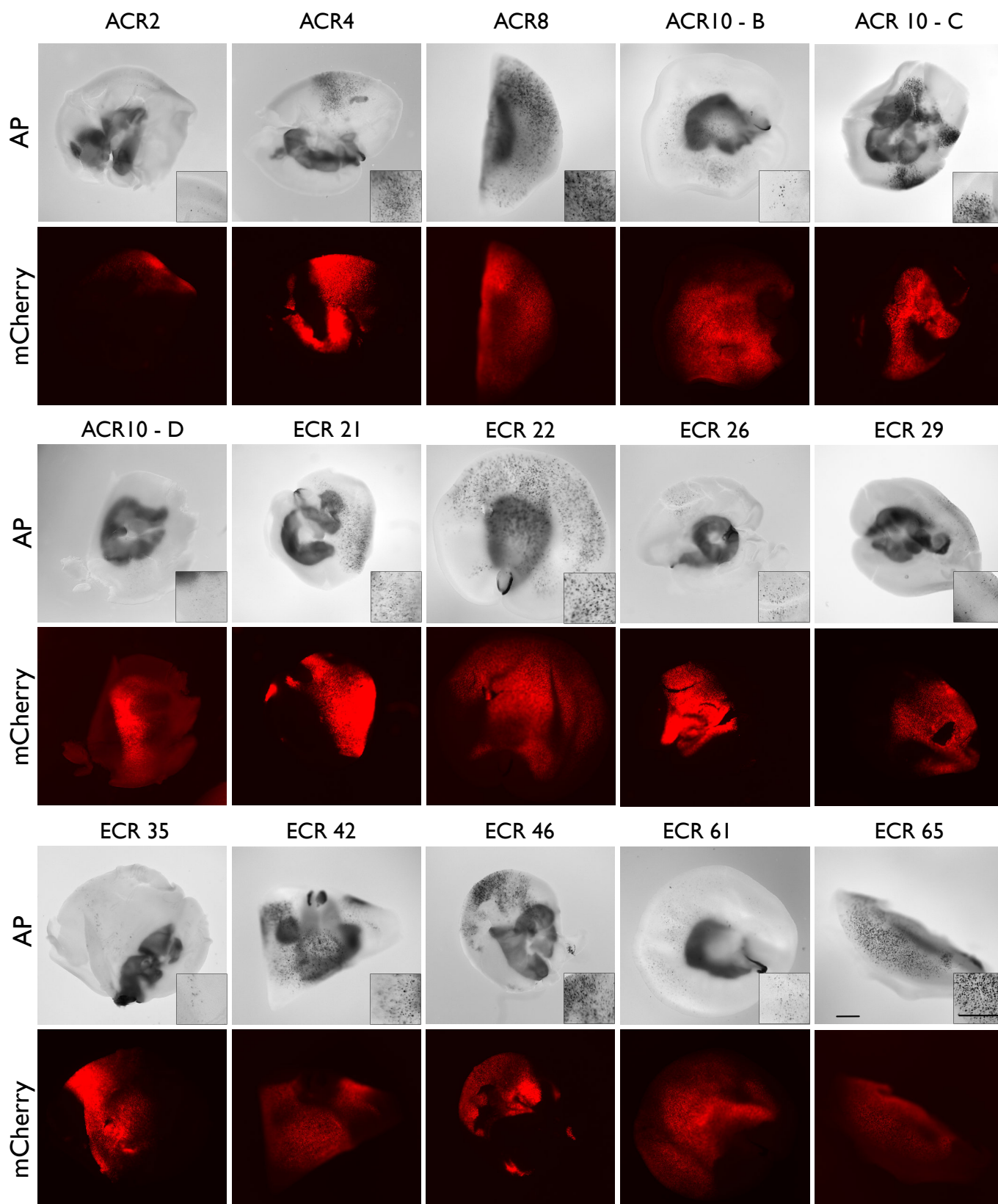

Supplement: Supplementary file 3 — Additional File 3. Regulatory elements active in E5 chick retinae. E5 chick retinae electroporated with Enhancer::AP plasmids and CAG:: mCherry plasmids and cultured for 1 day prior to alkaline phosphatase assay. Shown are the AP reporter signal on top and the mCherry signal on bottom. Insets in AP panels show zoomed in areas of reporter activity. Scale bar in last panel represents 500 μm and applies to all. [file 13064_2020_142_MOESM3_ESM.pdf]

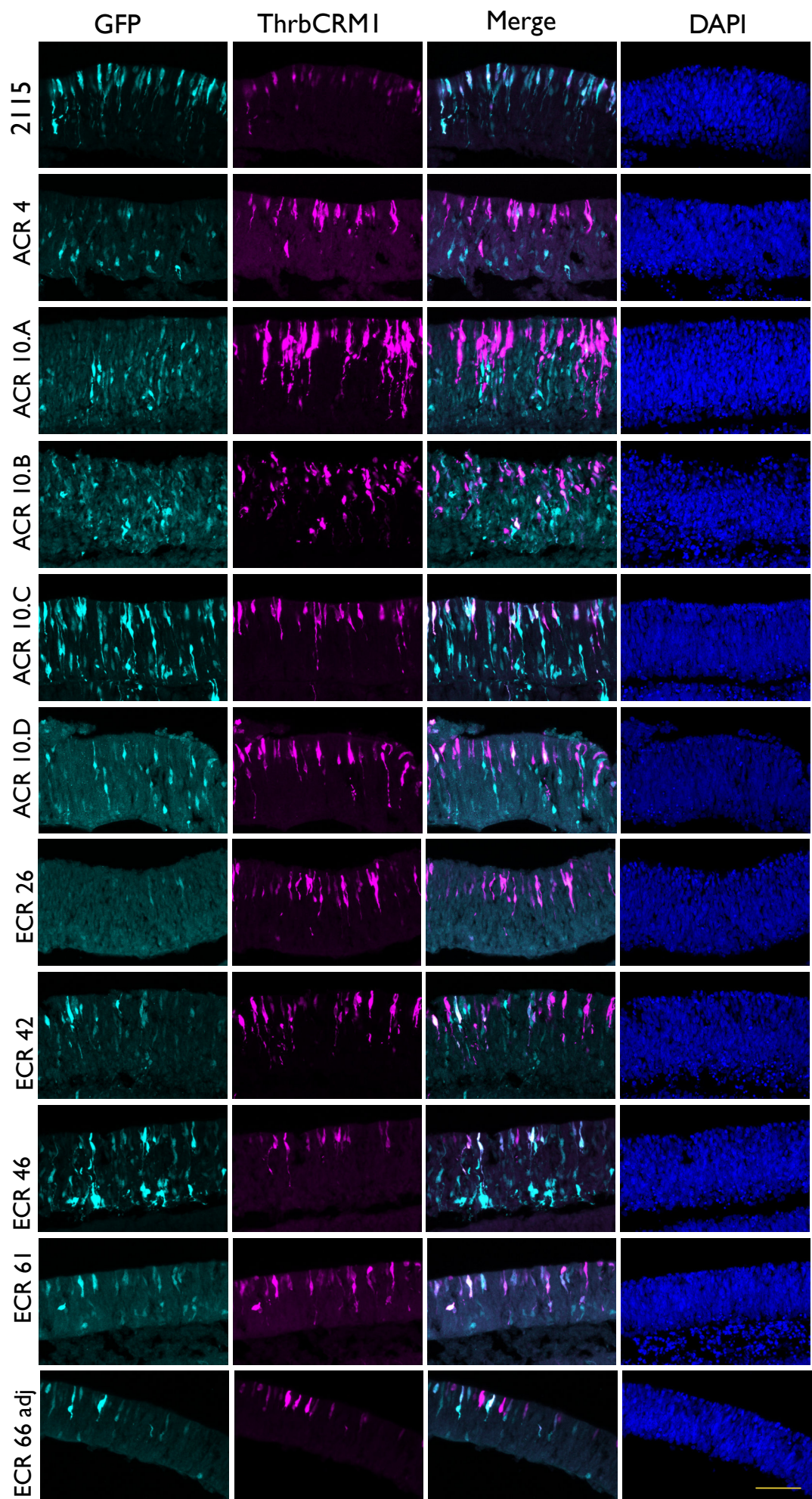

Supplement: Supplementary file 4 — Additional File 4. Overlap between ThrbCRM1 activity and activity of eleven candidate enhancers. E5 chick retinae were electroporated with enhancer::GFP (cyan) constructs as well as ThrbCRM1::AU1 (magenta) constructs and cultured for 18–22 h prior to antibody staining with GFP, AU1 and DAPI (nuclei) to determine which enhancers marked the same cell population as ThrbCRM1. Scale bar in last panel represents 50 μm and applies to all. [file 13064_2020_142_MOESM4_ESM.pdf]

GFP

Bgal

DAPI

ECR 42

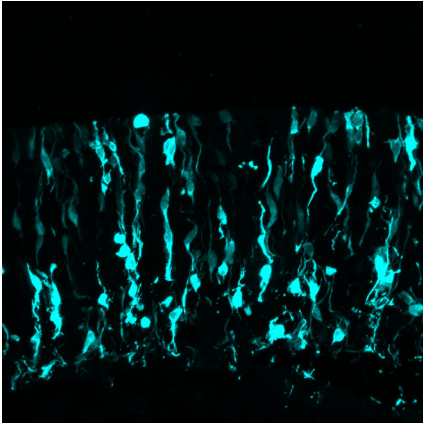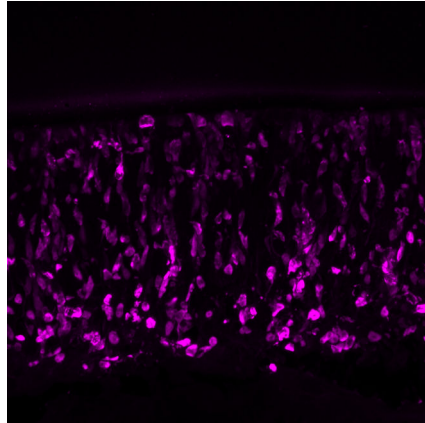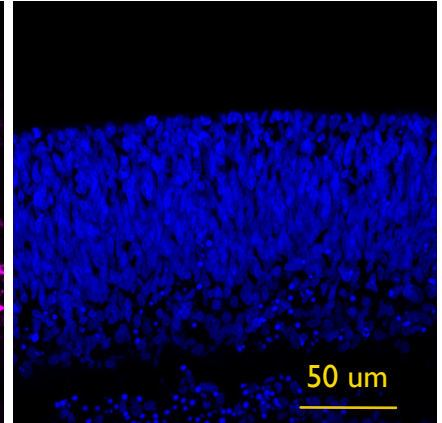

ACR 2

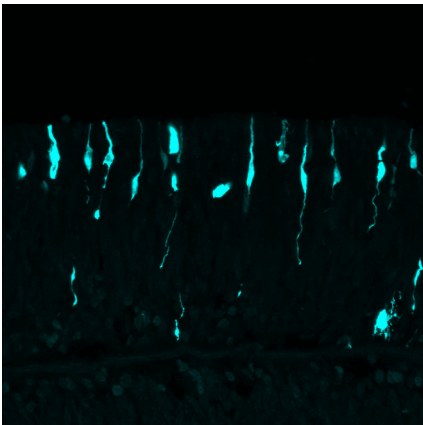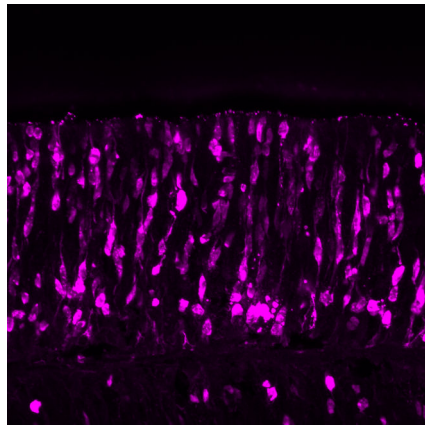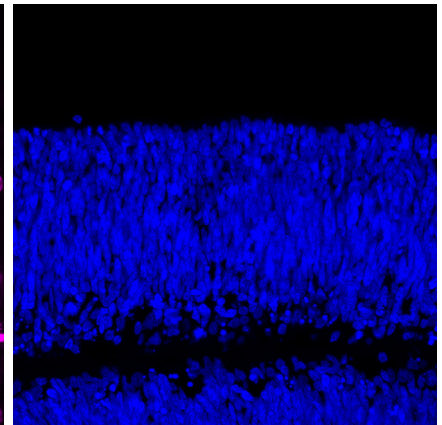

Supplement: Supplementary file 5 — Additional File 5. Lineage tracing of regulatory elements reveals range in specificity. ACR2::PhiC31 and ECR42::PhiC31 were electroporated into E5 chick retinae with a PhiC31 GFP responder plasmid and CAG::Bgal and cultured for two days before harvest and staining with GFP to label cells with a history of PhiC31 expression and Bgal to label all electroporated cells. Scale bar in top right panel represents 50 μm and applies to all. [file 13064_2020_142_MOESM5_ESM.pdf]

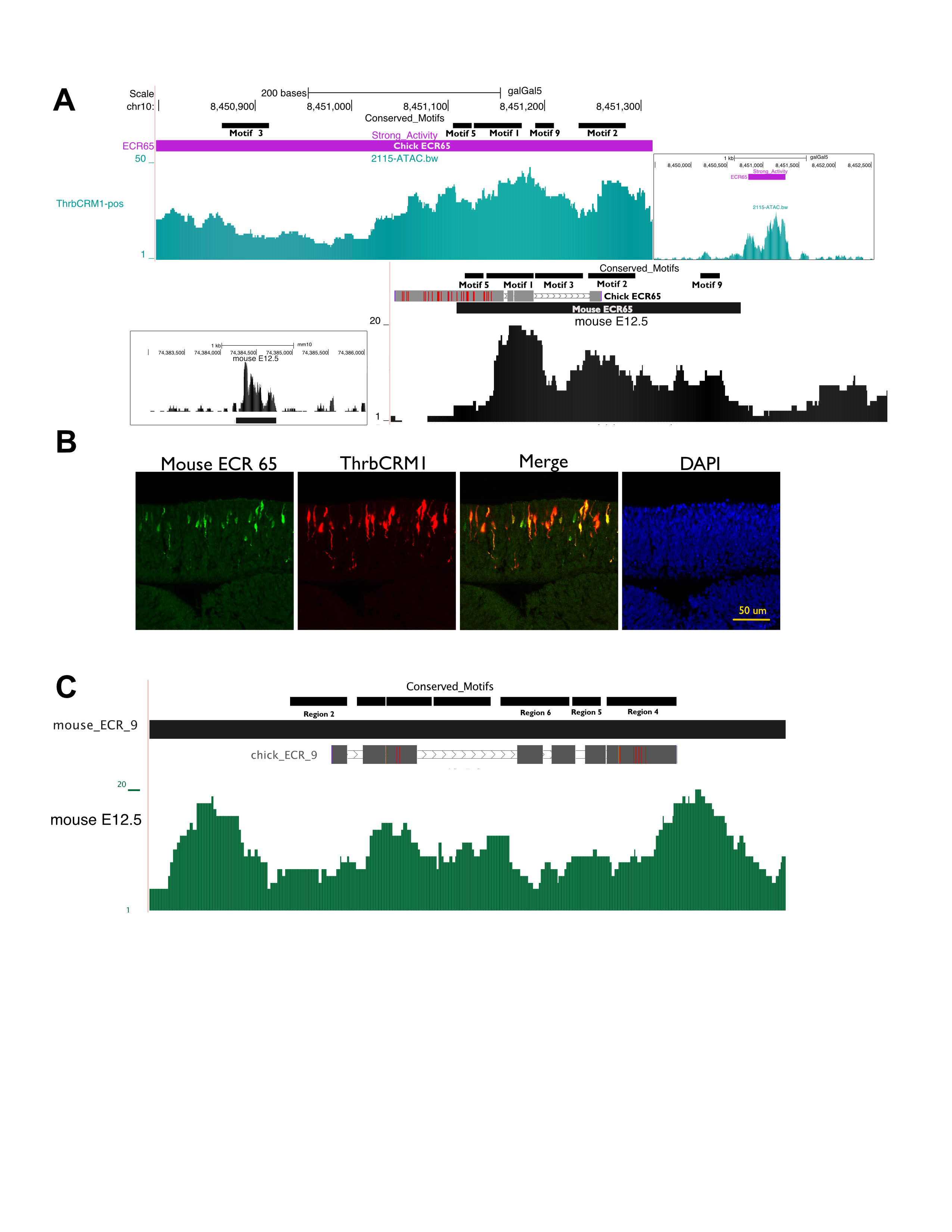

Supplement: Supplementary file 6 — Additional File 6. Conservation of sequence, chromatin state and function of ECR65 and ECR9. (A) The entirety of chick ECR65 (purple bar) aligns to open chromatin in the chick genome. The homologous mouse sequence (grey bar with red lines) only partly aligns to the open chromatin region in the mouse. Mouse ECR65 (long black bar) is a longer region of open chromatin. Regions 2 and 6 (small labelled black bars) are conserved between both Mouse ECR65 and Chick ECR65. Insets show zoomed out genomic area to include surrounding closed chromatin. (B) Mouse ECR65::GFP was electroporated into E5 chick retina along with ThrbCRM1::AU1 and cultured for 18–22 h before harvest and immunohistochemistry. Retinae were stained for GFP, AU1 and DAPI to examine overlap between GFP and AU1. Scale bar shown in last panel represents 50 μm and applies to all. (C) Chromatin accessibility at the ECR9 region in the mouse E12.5 retina. The thick black bar depicts the mouse ECR9 region, the grey bars represent the regions of homology to the chicken, and the thin black bars represent motifs identified in the mouse ECR9 sequence. [file 13064_2020_142_MOESM6_ESM.tiff]

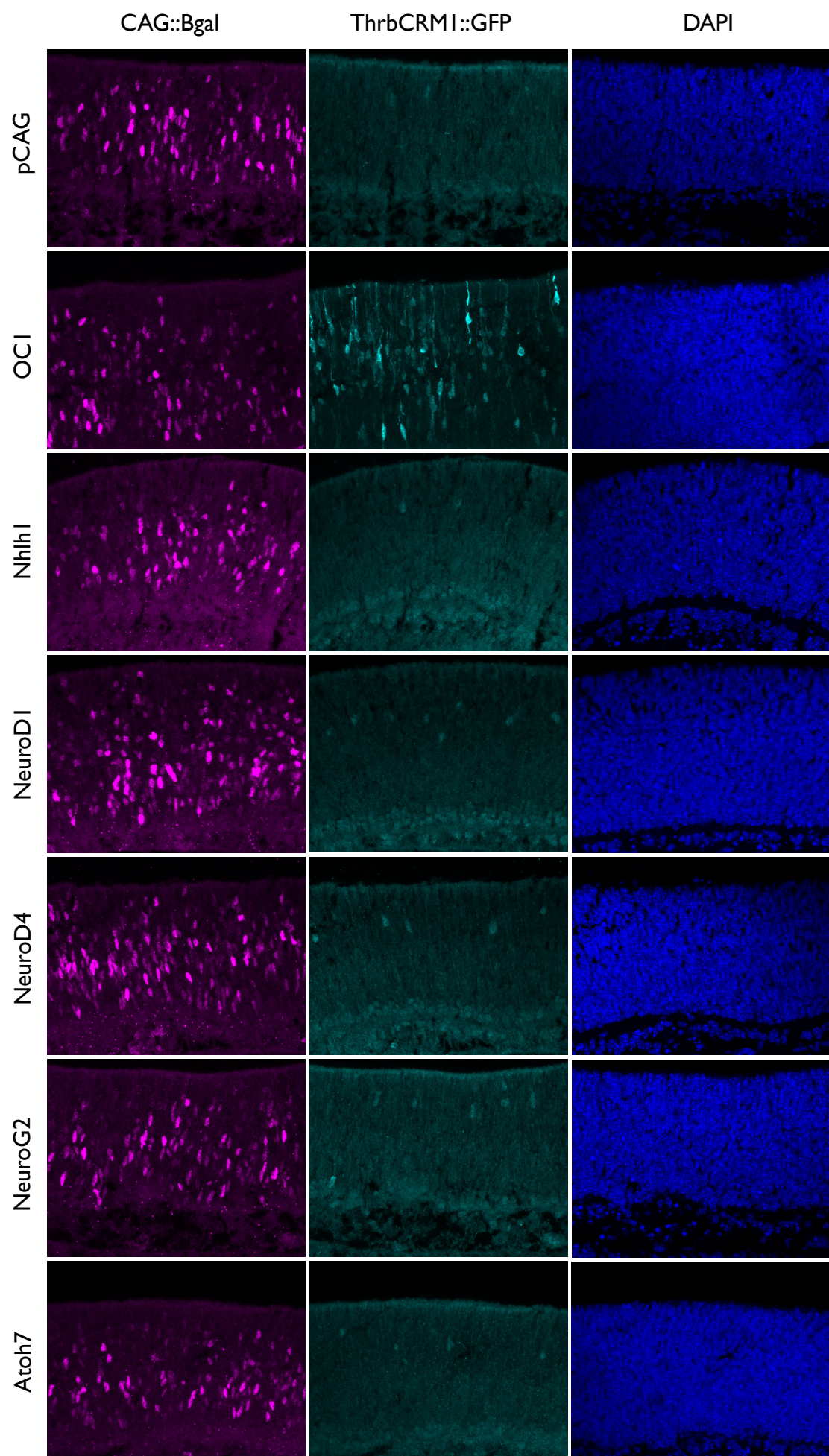

Supplement: Supplementary file 9 — Additional File 9. Candidate bHLH factors are not sufficient to induce ectopic ThrbCRM1 activity in the mouse postnatal retina. P0 mice retinae were electroporated with CAG::Bgal (magenta), ThrbCRM1::GFP (green) and the five candidate bHLH factors under the control of CAG. An empty CAG plasmid served as the negative control and CAG::OC1 as a positive control. Retinae were cultured for two days prior to harvest and staining with Bgal, GFP and DAPI. [file 13064_2020_142_MOESM9_ESM.pdf]

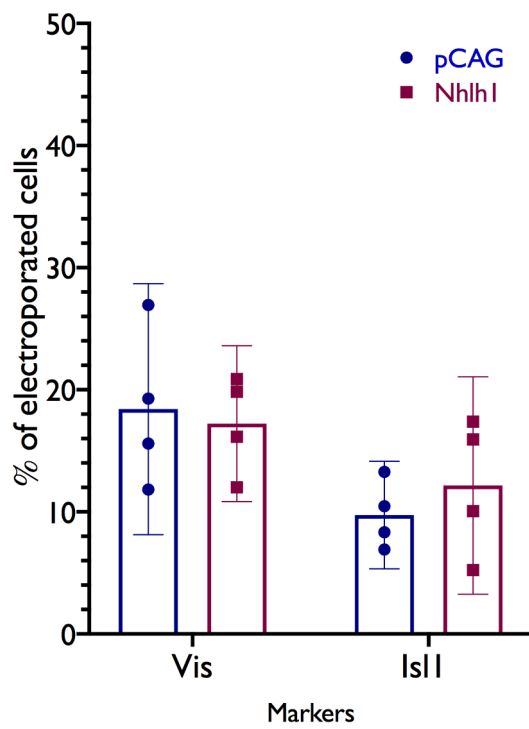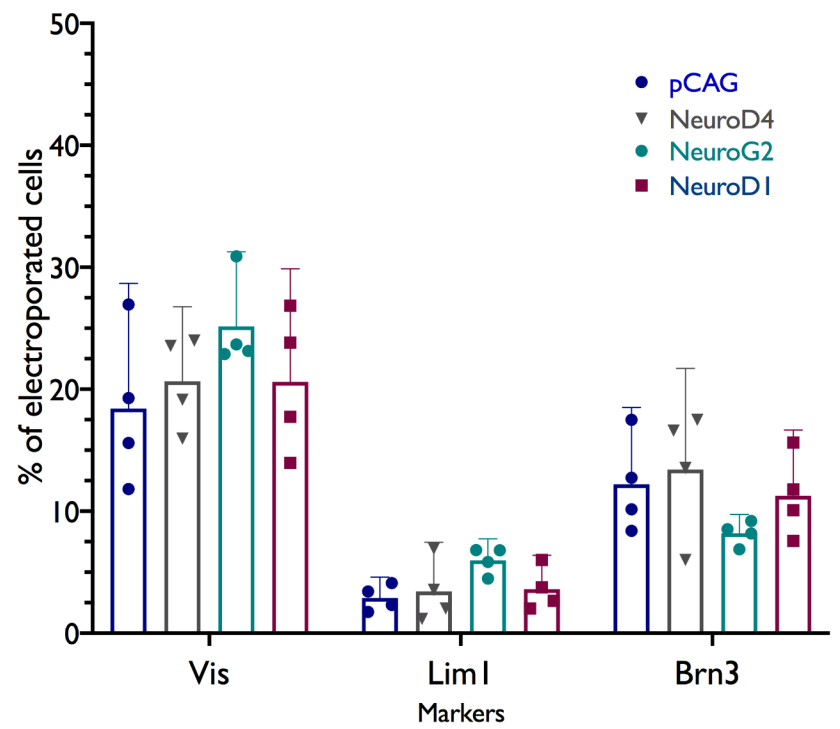

Supplement: Supplementary file 10 — Additional File 10. Individual bHLH factors are not sufficient to increase numbers of early retinal cell types. E5 chick retinae were electroporated with CAG::bHLH constructs and CAG::Bgal as an electroporation control and cultured for two days before being processed for immunohistochemistry. Retinal sections were stained with DAPI (nuclei), Bgal (electroporated cells) and cell-type specific markers. Percentages were calculated using the number of cells marked by each factor out of the total number of Bgal(+) cells. Error bars represent 95% confidence intervals. Each point represents a biological replicate. [file 13064_2020_142_MOESM10_ESM.pdf]
