# Supplementary material for: Cis-regulatory analysis of Onecut1 expression in fate-restricted retinal progenitor cells
Source: Neural Dev. 2020 Mar 19;15:5. doi: 10.1186/s13064-020-00142-w (PMC7082998; doi:10.1186/s13064-020-00142-w)
Supplement: Supplementary file 2 — Additional File 2. ID and genomic coordinates of all tested sequences in the galGal5 chick genome assembly, with the exception of ECR9 which is in the mm10 mouse genome assembly. [file 13064_2020_142_MOESM2_ESM.pdf]

Enhancer	Genomic Coordinates
**ECR1**	chr10: 8663469-8664114
**ACR1**	chr10: 8385494-8386148
**ECR2**	chr10: 8655229-8657051
**ACR2**	chr10: 8384185-8385075
**ECR_X**	chr10: 8650489-8651719
**ACR3**	chr10: 8375625-8376231
**ACR4**	chr10: 8514487-8515384
**ECR5**	chr10: 8636448-8636927
**ACR5**	chr10: 8512198-8513015
**5adj**	chr10: 8637057-8637759
**ECR6**	chr10: 8635344-8635535
**ACR6**	chr10: 8492309-8492485
**ECR7**	chr10: 8634455-8634704
**ACR7**	chr10: 8450103-8450313
**ECR8**	chr10: 8632688-8633007
**ACR8**	chr10: 8394607-8395238
**ECR9**	chr9:74846723-74847170
**ACR9**	chr10: 8393675-8394030
**ECR10**	chr10: 8631067-8631367
**ACR10.A**	chr10:8675208-8675805
**ACR10.B**	chr10:8675786-8676296
**ACR10.C**	chr10:8676249-8676699
**ACR10.D**	chr10:8676652-8677308
**ECR11**	chr10: 8625901-8626415
**ACR11**	chr10: 8642720-8643265
**11adj**	chr10: 8624685-8625065
**ECR_A**	chr10: 8622011-8622605
**ECR12**	chr10: 8616893-8617477
**12adj**	chr10: 8615429-8616540
**ECR14**	chr10: 8612329-8612808
**14adj**	chr10: 8613306-8615161
**ECR15**	chr10: 8607507-8609556
**ECR17**	chr10: 8604723-8605057
**ECR18**	chr10: 8602677-8603712
**ECR19**	chr10: 8599411-8599875
**ECR20**	chr10: 8595596-8596293
**ECR21**	chr10: 8594202-8594551
**ECR22**	chr10: 8593808-8594297
**ECR24**	chr10: 8584888-8585254
**ECR25**	chr10: 8584888-8585254
**ECR26**	chr10: 8576532-8577086
**ECR27**	chr10: 8576893-8577385
**ECR28**	chr10: 8577272-8577677
**ECR29**	chr10: 8569146-8569350

**ECR30**	chr10: 8567124-8567852
**ECR31**	chr10: 8563261-8563496
**ECR32**	chr10: 8563261-8563496
**ECR33**	chr10: 8552799-8553199
**ECR34**	chr10: 8552380-8552892
**ECR35**	chr10: 8545908-8546480
**ECR36**	chr10: 8542616-8545100
**ECR37**	chr10: 8539165-8539457
**ECR38**	chr10: 8535862-8536250
**ECR39**	chr10: 8535401-8535717
**ECR40**	chr10: 8531924-8532238
**ECR41**	chr10: 8531318-8531625
**ECR42**	chr10: 8528440-8528938
**ECR43**	chr10: 8526952-8526952
**ECR44**	chr10: 8521966-8522213
**ECR45**	chr10: 8521266-8522058
**ECR46**	chr10: 8515926-8516305
**ECR47**	chr10: 8507915-8508786
**ECR48**	chr10: 8505975-8506397
**ECR49**	chr10: 8501851-8502327
**49adj**	chr10: 8502221-8502639
**ECR50**	chr10: 8500530-8501106
**ECR51**	chr10: 8495478-8496218
**ECR52**	chr10: 8490472-8490869
**ECR53**	chr10: 8489091-8489919
**ECR54**	chr10: 8486341-8486607
**ECR55**	chr10: 8481081-8481361
**ECR56**	chr10: 8480144-8480410
**ECR57**	chr10: 8479342-8479641
**ECR58**	chr10: 8478198-8478383
**ECR59**	chr10: 8476275-8476779
**ECR60**	chr10: 8472598-8472844
**ECR61**	chr10: 8469909-8470514
**ECR62**	chr10: 8459873-8460361
**ECR63**	chr10: 8457431-8457602
**ECR64**	chr10: 8453886-8454273
**ECR65**	chr10: 8450796-8451312
**ECR66**	chr10: 8447307-8447542
**66adj**	chr10: 8446729-8447361
**ECR67**	chr10: 8444225-8444787
**ECR68**	chr10: 8442354-8442820
**ECR69**	chr10: 8436831-8437103

**ECR70**	chr10: 8432829-8433099
**70adj**	chr10: 8433359-8433724
**ECR71**	chr10: 8427766-8428649
**ECR72**	chr10: 8422264-8422774
**ECR73**	chr10: 8421785-8422274
**ECR74**	chr10: 8419319-8420332
**ECR75**	chr10: 8414515-8414968
**ECR76**	chr10: 8412870-8413389
**ECR77**	chr10: 8406698-8407162
**ECR78**	chr10: 8645415-8645691
