# Supplementary material for: Cis-regulatory analysis of Onecut1 expression in fate-restricted retinal progenitor cells
Source: Neural Dev. 2020 Mar 19;15:5. doi: 10.1186/s13064-020-00142-w (PMC7082998; doi:10.1186/s13064-020-00142-w)
Supplement: Supplementary file 7 — Additional File 7. Sequence alignments of ECR9 and ECR65 mouse, chicken and human homologous sequences. Asterisks below nucleotides denote conservation. Labelled black arrows demarcate boundaries of Motifs or Regions that were deleted in Fig. 4. ECR65 Region 3 and ECR65 Region 5 share a boundary. All deletions are directional as shown in Fig. 4. Mutated bHLH sites are shown below full alignments, highlighted in blue. [file 13064_2020_142_MOESM7_ESM.pdf]

```
                                                Region 1
ECR9_chick  ------------------------------------------------------------
ECR9_mouse  TTGCACTTCCTGTGGATTAGTGAACAGATCTCTTTATAGGACTACATCTGTCTCTCTCTT
ECR9_human  ------------------------------------------------------------

ECR9_chick  --------------------AGGGTAGGGCTTCCCAGGAAGGGTTAAGGCTTTTCAGGCC
ECR9_mouse  CCACAATCTGTCCTACCCTAGGGGGAGGGCTGGCGGGAAAGGGTTAAAGGTTTTCTGGTC
ECR9_human  ------------------------------------GAAAGGGTTAAAGGTTCTCTGGTC
                                                * ********* * ** ** ** *
                              Region 2
ECR9_chick  AAAATCCTCCAACAGAAGGCAGGGTCAGCCTCGGAGCTGAAATCTAAGTGCTAATGACTG
ECR9_mouse  GAAATAGCCCAACAGA---AGGGGCCCGGCCCAGAGCTGAAATCTAAGT-CTAATGACCG
ECR9_human  AAAATAGCCCAACAGA---AGGAGCTGGGCCCAGAGCTGAAATCTAAGTGCTAATGACCG
             ****   ********     * *   * * * **************** ******** *

ECR9_chick  ATGCCCTTCACCCCTCCGTCGCCTCCAAAGGCGACTGCCCTGGATGAGC----CGGG---
ECR9_mouse  CTGCCCTTCACCTCGCTGCTGTCCACAGGCCGGACCTGCGGCCCGGTGGGGGCCGGGACC
ECR9_human  TTGCCCTTCACCCTACTGCTGTCCACTGGCCGGACCTGCGGCCGGGTGGGGCCCCGGACT
             ***********   * *  * *  *      ***   *      * *     * **
                     Region 6
ECR9_chick  ------------GCCGGAGAGCATTTAACATTTGAACTTCTTCACGTCTGCGAGGCAGCA
ECR9_mouse  AGGACAGGACACAATGTTCTTCGTTTAACATTTGAACTTC---TCCTATCCGAGGCAGCA
ECR9_human  GGGAGAGGGCACAATATTCTGCATTTAACATTTGAACTTC---TCCTATCCGAGGCAGCA
                                 * *****************    * * * **********
              Region 5                                Region 4
ECR9_chick  GGCCGTTGTGCGCGGGGT-CGCTGACCCCCAGCCCCGCTCCCAGCCCCTCAGCTGCCTC
ECR9_mouse  GGCCGTTTTGTCCTGGGGTCGCTGAC----------CCTCGCTCCCCCATCAGCTGCCCT
ECR9_human  GGCCGTTTTGTCCTGGGGTCGCTGAC----------CCTGGCTCCCCCATCAGCTGCCCT
            ******* **  * ***  *******           **  *   *** *********
                                      Region 3
ECR9_chick  CCGGCCCTAATTGAATGACAGATGGTCAGCGGCAGCGAGAGGGCAGGAGCCGGCCCCGCG
ECR9_mouse  GAAACCTTAATTGAATGACAGATGGTCATTTTCGGGGGCGCCAGAAGCTGCGCTGGCCAG
ECR9_human  GAAACCTTAATTGAATGACAGATGGTCAT-------------------------------
                ** *********************

ECR9_chick  CCCCCAGCACTGCTGCCCACGCGCCCCAGGGCCGCCTCGGCCGCCCACTAAGGGACGGGG
ECR9_mouse  TGG-AGGGGCTCAGGCCGAGAAGGCCTTGCTTAGGCTCTGCCTGCT--------------
ECR9_human  ------------------------------------------------------------
```

ECR9 bHLH Site 4

```
GGCCCTAATTGAATGACAGATGGTCAGC
AACCTTAATTGAATGACAGATGGTCATT
AACCTTAATTGAATGACAGATGGTCAT-
  ** *********************
```

ECR9 bHLH Site 3

```
CCGCTCCCCAGCCCCTCAGCTGCCTC
--CCTCGCTCCCCCATCAGCTGCCCT
--CCTGGCTCCCCCATCAGCTGCCCT
   **  *   *** *********
```

```
ECR65_chick  TTTCCATGCATCAGCTGCTATTGTATGACCATACATAATTGGGTCTAAGTGCTTACATCA
ECR65_mouse  ------------------------------------------------------------
ECR65_human  ------------------------------------------------------------

ECR65_chick  GATGCCTATTATGTGAAATTAATTTTTGGAACATATGCTGTTGTATTTATACCATTGTTG
ECR65_mouse  ------------------------------------------------------------
ECR65_human  ------------------------------------------------------------

ECR65_chick  TTTCATGCTAGATTAATTCTTCAAGGGTTATAATCACATGACACTTATCATTTTAATTAT
ECR65_mouse  ------------------------------------------------------------
ECR65_human  ------------------------------------------------------------
                              Region 1
ECR65_chick  GCCTCTTTAGTTGCAACATATGACAGCTTTTGACTTCTCACCCTCTCCTTCTGCATGTGT
ECR65_mouse  -------------------------------------------------TTGTATTGTGT
ECR65_human  -------------------------------------------------TTGTATTGTGT
                                                              *     *****
                              Region 2
ECR65_chick  GACAGATAAAATTATTAATAAATGCTTGTTTATTATGTAGTTATGCTGTACATGTCCACA
ECR65_mouse  GACCAATGAGATTATTAATAAATGCCTTTTCATTGTGTATCCAGGCTGTAGGTGCCCACG
ECR65_human  GACCAATGAGATTATTAATAAATGCTTTTTTATTATGTAGTCATGCTGTAGGTGCCCACA
             ***  ** * *************** * ** *** ****   * ******  ** ****
                                                                  Region 3/5
ECR65_chick  GGGTGTAGGTTGGCCTTGTGTATTTTACCCAGAGTACAGTGTCAGCAGAGCCTTTGTGGT
ECR65_mouse  GGGTATGGGGCAGCCTCCTGTATTTTATTCTGGGTGCAGTGTCAGCAGGACCTTGTGGAT
ECR65_human  GGGTATGGGCTGGCCTCATGTATTTTATTCTGGGTGCAGTGTCAGCAGGGCCTTGCGGAT
             **** * **   ****  *********  * * ** ************  ****   * *
                                                                    Region 4
ECR65_chick  TTGCTAATGAGCTAAATCCCACTAACACCACACACACTCTAAACTGCCAAAAGCCCTTTA
ECR65_mouse  TTGCTAATGAGCTAAATCCGGTTAGTGCCACACGCCCTGGATGGC--CAGAGATCTTTTA
ECR65_human  TTGCTAATGAGCTAAATCCGGTTAGTGCCACATGCCCTGGACTGC--CAGAGATCTTTTA
             *******************   **   *****  * **  *      ** *   * ****

ECR65_chick  CACATTCAAACACATACAAAGACTCAGCTGGGTCCTTTGTATAAAAGATTAACTCTTTAT
ECR65_mouse  AATACATCATTGCTCACAGAGACTCAGCTGGACTCAGGCAGAGAGAGATTAACTCCTCAT
ECR65_human  AATACATCATTGCTCTCAGAGACTCAGCTGGGTTCAGTATGAGAGAGATTAACTTCTCAT
              * *    *   *   ** ************   *        * *********  * **

ECR65_chick  TCCAATTAAAGATGAGCTGGCCTGGCTTGGTCCAA-------------------------
ECR65_mouse  TCCAATTAGCAAAGAGTCCGACTGTTGCATGT-GGCTTGCTTCTGTAGCCCCAAACCCGG
ECR65_human  TCCAATTAACAGAGAGTCATACTGCTGGAGAGAGGGTTGCTTATGTAGCCCCAAACCTAG
             ********     ***     ***
```

ECR65 bHLH site

```
TACAAAGACTCAGCTGGGTCCTT
CACAGAGACTCAGCTGGACTCAG
CTCAGAGACTCAGCTGGGTTCAG
  ** ************   *
```
