# Supplementary material for: Cis-regulatory analysis of Onecut1 expression in fate-restricted retinal progenitor cells
Source: Neural Dev. 2020 Mar 19;15:5. doi: 10.1186/s13064-020-00142-w (PMC7082998; doi:10.1186/s13064-020-00142-w)
Supplement: Supplementary file 8 — Additional File 8. Unscaled values from deletion, mutation, and overexpression experiments (A) ECR65 activity from deletions and mutations corresponding to Fig. 4. SP52 and NJ849 refer to two different orientations of ECR65::GFP. (B) ECR65 activity with empty pCAG vector, corresponding to Fig. 5. (C) ECR9 activity from deletions and mutations, corresponding to Fig. 4. NJ1140 and NJ1142 refer to two different orientations of ECR9. (D) ECR9 activity with the empty pCAG vector, corresponding to Fig. 5. (E,F) Mutations of ECR65 and ECR9 with different mutant sequences, corresponding to Fig. 4. Error bars represent 95% confidence interval. Each depicted point represents a biological replicate. [file 13064_2020_142_MOESM8_ESM.pdf]

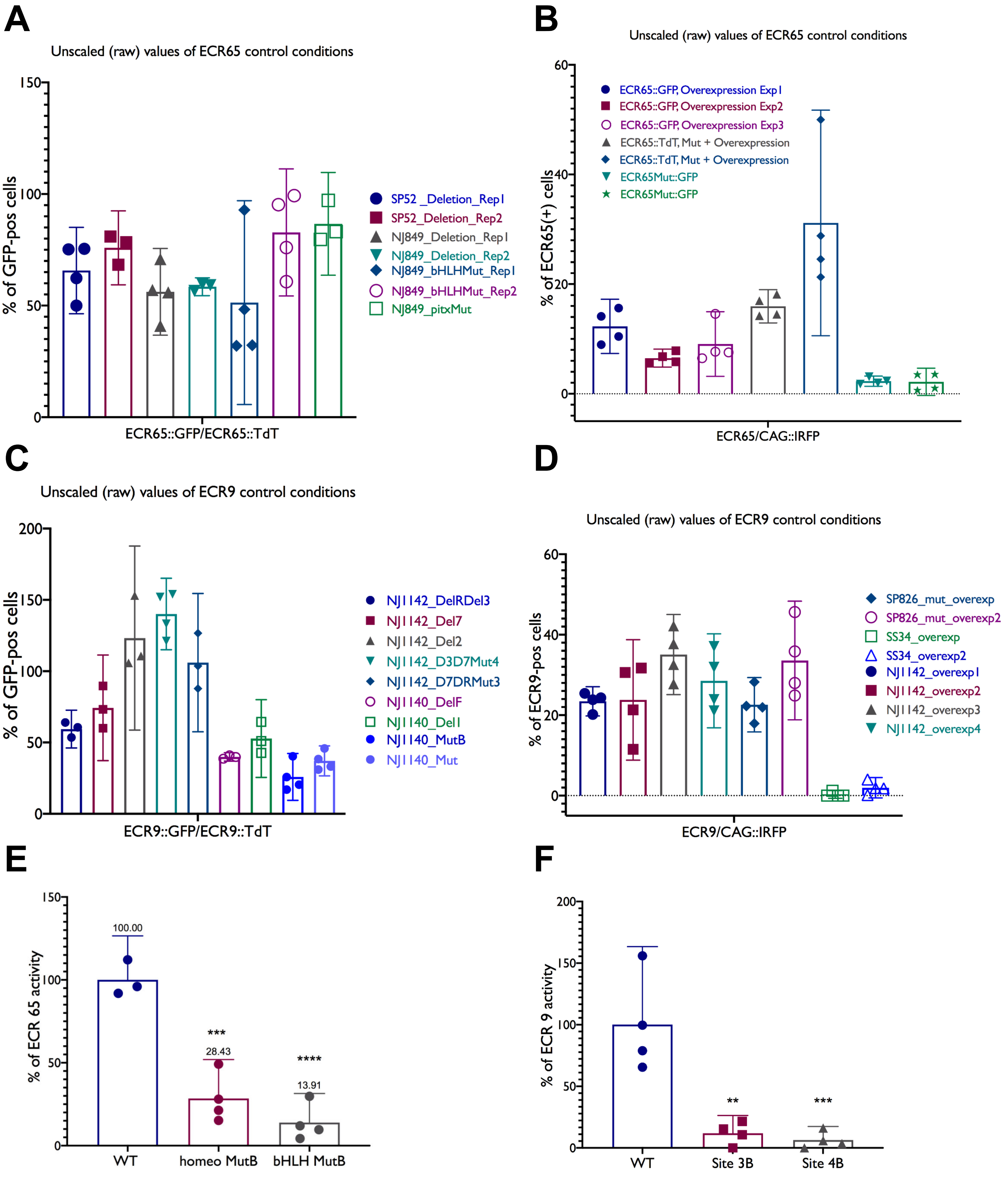
A
Unscaled (raw) values of ECR65 control conditions
% of GFP-pos cells
150
100
50
0
ECR65::GFP/ECR65::TdT
SP52 _Deletion_Rep1
SP52_Deletion_Rep2
NJ849_Deletion_Rep1
NJ849_Deletion_Rep2
NJ849_bHLHMut_Rep1
NJ849_bHLHMut_Rep2
NJ849_pitxMut
B
Unscaled (raw) values of ECR65 control conditions
% of ECR65(+) cells
60
40
20
0
ECR65/CAG::IRFP
ECR65::GFP, Overexpression Exp1
ECR65::GFP, Overexpression Exp2
ECR65::GFP, Overexpression Exp3
ECR65::TdT, Mut + Overexpression
ECR65::TdT, Mut + Overexpression
ECR65Mut::GFP
ECR65Mut::GFP
C
Unscaled (raw) values of ECR9 control conditions
% of GFP-pos cells
200
150
100
50
0
ECR9::GFP/ECR9::TdT
NJ1142_DelRDel3
NJ1142_Del7
NJ1142_Del2
NJ1142_D3D7Mut4
NJ1142_D7DRMut3
NJ1140_DelF
NJ1140_Del1
NJ1140_MutB
NJ1140_Mut
D
Unscaled (raw) values of ECR9 control conditions
% of ECR9-pos cells
60
40
20
0
ECR9/CAG::IRFP
SP826_mut_overexp
SP826_mut_overexp2
SS34_overexp
SS34_overexp2
NJ1142_overexp1
NJ1142_overexp2
NJ1142_overexp3
NJ1142_overexp4
E
% of ECR 65 activity
150
100
50
0
100.00
***
28.43
****
13.91
WT
homeo MutB
bHLH MutB
F
% of ECR 9 activity
200
150
100
50
0
**
***
WT
Site 3B
Site 4B
